# Obesity, Diabetes Mellitus, and Vascular Impediment as Consequences of Excess Processed Food Consumption

**DOI:** 10.7759/cureus.28762

**Published:** 2022-09-04

**Authors:** Susmita Sinha, Mainul Haque

**Affiliations:** 1 Physiology, Khulna City Medical College and Hospital, Khulna, BGD; 2 Pharmacology and Therapeutics, National Defence University of Malaysia, Kuala Lumpur, MYS

**Keywords:** blood vessels, phosphate, preservatives, food additives, freshly, oven to table, precooked food, prepackaged food, unhealthy food, junk food

## Abstract

Regular intake of ready-to-eat meals is related to obesity and several noninfectious illnesses, such as cardiovascular diseases, hypertension, diabetes mellitus (DM), and tumors. Processed foods contain high calories and are often enhanced with excess refined sugar, saturated and trans fat, Na^+ ^andphosphate-containing taste enhancers, and preservatives. Studies showed that monosodium glutamate (MSG) induces raised echelons of oxidative stress, and excessive hepatic lipogenesis is concomitant to obesity and type 2 diabetes mellitus (T2DM). Likewise, more than standard salt intake adversely affects the cardiovascular system, renal system, and central nervous system (CNS), especially the brain. Globally, excessive utilization of phosphate-containing preservatives and additives contributes unswervingly to excessive phosphate intake through food. In addition, communities and even health experts, including medical doctors, are not well-informed about the adverse effects of phosphate preservatives on human health. Dietary phosphate excess often leads to phosphate toxicity, ultimately potentiating kidney disease development. The mechanisms involved in phosphate-related adverse effects are not explainable. Study reports suggested that high blood level of phosphate causes vascular ossification through the deposition of Ca^2+^ and substantially alters fibroblast growth factor-23 (FGF23) and calcitriol.

## Introduction and background

Current studies regarding the consumption of ready-prepared foods and their consequences reliably reported a significant correlation between obesity, several noninfectious illnesses (noncommunicable diseases (NCDs)), cardiovascular diseases, hypertension, several types of malignancies, and many more [1-5]. For the last few decades, obesity has appeared as a significant public health issue globally [6]. One US study reported that 35.65% of the adult population was obese in 2009-2010, and no statistically substantial transformation was observed when equated with 2003-2008 [5]. Obesity linked with high consumption of ready-to-eat meals equally exists in low- and middle-income countries (LMICs) and high-income countries (HICs) [7-12]. The World Health Organization (WHO) stated in 2020 that “39% of adults aged 18 years and over were overweight in 2016, and 13% were obese.” Furthermore, the WHO outlined overweight as a “body mass index (BMI) more than or equivalent to 25, and obesity as a BMI above or equally 30” [13]. Additionally, it has been apprised that by 2030, 38% and 20% of the overall global grownup people will be overweight and obese, respectively [14]. The current manuscript will elaborate on the relation of fast-food consumption with obesity and its cardiovascular relevance. Processed food contains a high quantity of calories. These foods often contain excess refined sugar, saturated and trans fat, Na^+^ and phosphate-containing taste enhancers, and preservatives [15,16]. Most processed food contains highly refined carbohydrates that alter insulin physiology and promote adipose tissue deposition [17,18]. Studies have revealed that extremely treated foodstuffs with extra lipid substances and sugar-sweetened beverages change neurobiological reward pathways involved in eating behaviors, taking over the brain’s emotional and motivational pathways and encouraging food cravings and excessive food intake [19-21].

Materials and methods

This review explains the probable processes relevant to processed food and obesity, linking diabetes mellitus (DM) with vascular impediments and implications. The literature search was based on electronic archiving through Google Scholar, ScienceDirect, PubMed, and ResearchGate. The listing of references of allied papers was checked to obtain additional literature. Keywords include diabetes, obesity, food additives, food preservatives, processed foods, ready-to-eat foods, phosphate toxicity, vascular calcification, cardiovascular diseases, renal impairment in phosphate toxicity, and mitochondrial dysfunction. Papers published earlier than 2000 and written in languages other than English were discarded, as well as those papers with full texts that are not available through interlibrary collaboration. Before inclusion in this study, a manual check of appropriate articles was conducted. Duplicate articles were removed carefully. After the separate evaluation and addition of the nominated pieces of literature, a supplementary discussion was conducted to sort out every doubt, issue, error, or bias concerning particular articles.

## Review

Brief portrayal regarding obesity and ultra-processed food

One recent British study revealed that eating ultra-processed food regularly statistically significantly increases the possibility of obesity [22]. The British National Diet and Nutrition Survey (2008-2016) revealed through model analysis that among British people who eat prepackaged food, the highest (¼) group was correlated with 1.66 kg/m^2^ greater BMI, 3.56 cm higher waist circumference (WC), and 90% elevated odds for living with obesity than the bottommost group [23]. Similarly, another Korean study revealed that women who derived 26.8% of the total calories of daily need from extremely processed food (top ¼) had 0.61 kg/m^2^ inflated body mass index, 1.34 cm raised WC, 51% high-level odds of the presence of obesity (BMI > 25 kg/m^2^; odds ratio (OR): 1.51; 95% confidence interval (CI): 1.14-1.99; p = 0.0037), and 64% greater odds of central obesity (men: WC ≥ 90 cm; women: WC ≥ 85 cm; OR: 1.64; 95% CI: 1.24-2.16; p = 0.0004) when compared with lowest ¼ eaters of unhealthy junk food [24]. In the same way, one recent Australian study reported that a diet containing a lion’s share of convenience food is statistically significantly (p ≤ 0.001) correlated with obesity. This study developed adjusted results and stated that those Australians who eat the highest ¼ ultra-processed food had statistically significantly (p > 0.05) more BMI and WC and had additional odds for obesity and abdominal obesity than individuals of the lowermost ¼ [25]. The US population who derived 74.2%-100% calories from ready-to-eat junk food was correlated with 1.61 parts of raised BMI, bigger WC, and central obesity than those who derived 0%-36.5% energy from similar convenience food [26]. A Brazilian study reported an almost comparable picture. The adjusted result said that Brazilians consume the highest portion of prepackaged unhealthy food, and a high BMI level increases the possibility of developing statistically important additional WC than individuals who consume a minor portion [27]. Therefore, it is globally well known that prepackaged junk foods, because of their high amounts of sugar, Na^+^, and fat, cause obesity and several NCDs [1,7,16,28-32].

A Brief Depiction Regarding Food Additives and Preservatives

Foodstuff additions (preservatives, artificial sweeteners, emulsifiers, dyes, and color) are commonly utilized throughout the globe [33-35] to increase a vendible quality by increasing aroma, color, texture, appearance, and taste [36]. These agents are frequently correlated with multiple health hazards with hypersensitive reactions, e.g., diarrhea, abdominal discomfort, and colicky pains (digestive); nervous hyperactivity, insomnia, and irritability; glucose intolerance in healthy human subjects (endocrine); and dysbiosis [37-40]. Commonly used food additives are cheap [41] and widely used in commercial food processing. The most commonly used food additives, including preservatives, are monosodium glutamate (MSG), phosphate, synthetic food coloring agent, NaNO_2_, high-fructose containing liquid, synthetic sweetening agent, C_23_H_23_FN_4_O_7_Zn (Irish moss), C_7_H_5_NaO_2_, trans-unsaturated fatty acids, bacterial polysaccharide, synthetic seasoning agent, fermenting fungus, NaCl, C₃H₆O₂, disodium pyrophosphate (Na_2_H_2_P_2_O_7_), benzene-based carboxylic acid (C_6_H_5_CO_2_H), C_4_H_6_NaO_4_-, and phosphorus and its congeners [42-44]. The currently FDA-approved food and color lists can be retrieved at https://www.fda.gov/food/food-ingredients-packaging/food-additive-listings and https://www.fda.gov/industry/color-additive-inventories/color-additive-status-list, respectively.

Monosodium Glutamate in Food and Health

MSG, a Na^+^ salt of α-amino acid (C_5_H_9_NO_4_), is one of the most widely utilized taste enhancers by food manufacturers throughout the globe. MSG gives an exclusive flavor to the foodstuffs termed “umami” or “savory taste,” unlike the four principal tastes sweet, sour, salty, and bitter [45]. Besides, food safety regulatory organizations think MSG ingestion to be harmless, although considerable preclinical and clinical research has called into question MSG health risk exclusively subsequent to chronic ingestion.

**Figure 1:**
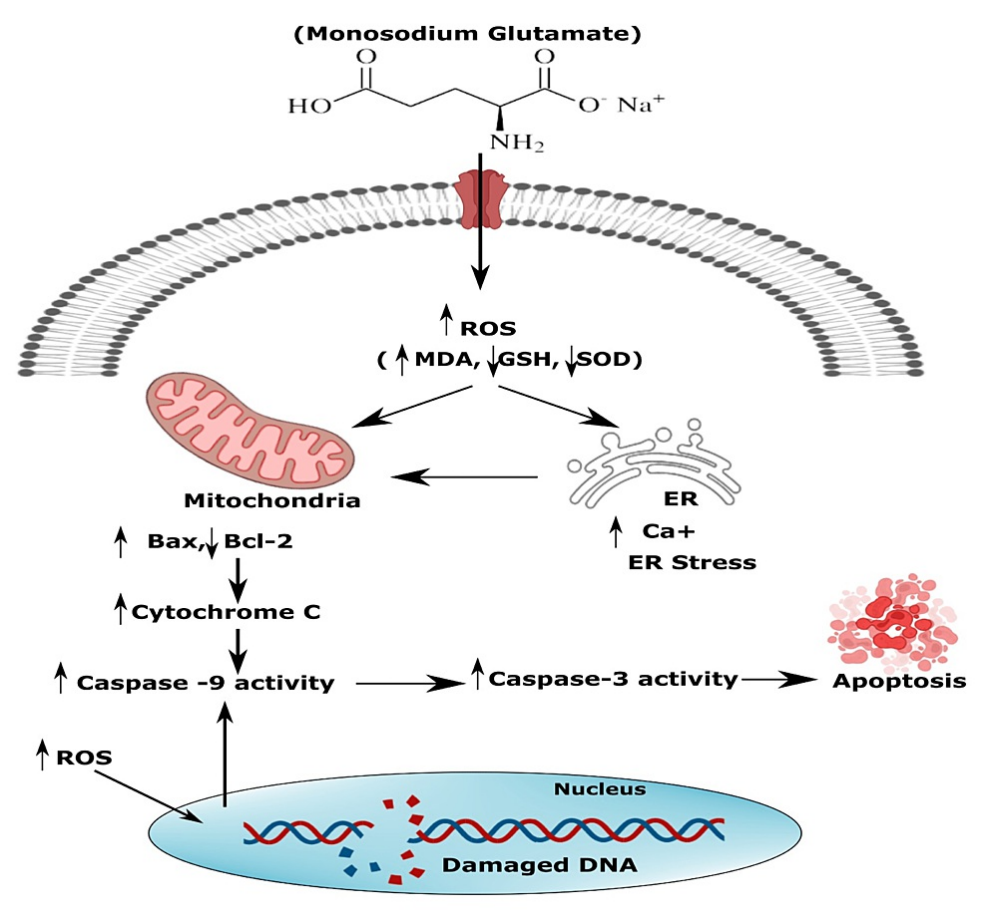
Molecular mechanics of cellular death provoked by MSG. MSG: monosodium glutamate; ROS: reactive oxygen species; MDA: malondialdehyde; GSH: glutathione; SOD: superoxide dismutase; ER: endoplasmic reticulum Image credit: Susmita Sinha

Glutamate has several physiological functions (Figure 1), such as acting as a substrate for energy creation in the intestinal epithelial cell lining, an intermediate in the cellular protein metabolic process, and the forerunner of important metabolites such as glutathione (GSH) (oxidative stress modulator) or N-acetyl glutamate (metabolic regulator) and also a central nervous system (CNS) excitatory neurotransmitter [46]. Glutamate receptors are expressed in other neuronal cells, including lymphocytes, thymocytes, and neutrophils [47]. An experimental study showed that MSG resulted in the development of obesity within six days because of a shoddy but insistent inflammatory condition, going along with pro-inflammatory cytokine step-up and suppressed adiponectin gene process, which is probably related to being overweight [48]. Moreover, MSG-induced enhanced strata of oxidative stress and extreme hepatic lipogenesis are encouragingly connected to obesity and T2DM [49]. Cellular hyperexcitability could be an essential contributing and pathogenic factor of cancer through the stimulation of voltage-gated Na^+^ passage, excitatory ligand glutamate, and non-neuronal excitative receptors [50].

Sodium Chloride (Salt) as Food Preservative

Salt (sodium chloride) has been used as a food preservative conventionally, limiting the development of foodborne pathogens and decomposition organisms by reducing water content. Na^+^ is an important nutrient for sustaining well-being when consumed moderately, and excess intake causes several health complications, such as cardiovascular disorders [51]. Bread, cheeses, processed meat, soy sauce, bacon, and spreads are the leading causes of high Na^+^ in modern foodstuffs [52]. NaCl is vital in ultimate taste enhancement, consistency, long shelf life, customer palatability, and germ-free and reasonable preparations of several treated meat foodstuffs [53]. In recent decades, preclinical, clinical, and epidemiological research have shown substantial and clinically important relations in collecting health-related problems for salt consumption above 4 g per day. Furthermore, high salt intake adversely affects vasculature, cardiovascular, renal, and central nervous systems, especially the brain [54].

Source of Phosphorus in Diet

Phosphorus is an essential micronutrient that originates predominantly from the phosphorus-comprising complex PO_4_^3-^ and is absorbed in the intestine from consumed food and food additives and circulated in the blood as inorganic phosphate (Pi). Again, plant sources (hard-shelled edible fruits, legumes, and whole grains) and animal sources (dairy, flesh, fish, and eggs) of phosphorus are available adequately as foodstuffs in nature. The natural phosphorus supply is in the crystalized state of carboxylic acid derivatives and hydroxyapatite [55,56]. Individuals who take vegetarian diets express reduced serum phosphorus echelons and declined fibroblast growth factor-23 (FGF23); additionally, plant-origin phosphorus is often associated with a salt of phytic acid (myo-inositol 1,2,3,4,5,6-hexakis dihydrogen phosphates), poorly assimilated (<40%) through our digestive tract [57]. However, studies showed that excess phosphate is one of the more important and dangerous reasons for cardiac problems and can even lead to death with or without negotiated renal function [58,59]. Elevated levels of PO_4_^3-^ are commonly linked with ossification of vessels, cardiovascular problems including left ventricular failure (LVF), defective endothelium function, and progression in declined kidney function [60].

Phosphate Homeostasis in Healthy Adults

The normal phosphorus level is 2.5-4.5 mg/dL (0.81-1.45 mmol/L). High serum levels of phosphorus in our system are described as plasma phosphate over 4.5 mg/dL due to illness or too much consumption of food [61]. Equilibrium in PO_4_^3-^ is regulated through the intestinal absorption of dietary sources, bony storage and release, and renal elimination. The principal endocrine control of the phosphate biotransformation process includes calcitriol, parathyroid hormone (PTH), and FGF23. In addition, about 60%-70% of dietetic phosphorus goes to the blood through the small gut, mostly through an energy-dependent Na/Pi symport [62]. Calcitriol excites gastrointestinal Na/Pi symport, thus augmenting PO_4_^3-^ absorption, but calcitriol controls only 30% of intestinal absorption [63].

Typically, the quantity of PO_4_^3-^ eliminated through the kidney usually equals that absorbed through the intestinal epithelial wall. Again, PTH decreased PO_4_^3-^ reabsorption from the proximal convoluted tubule by inferring Na/Pi cotransporters, although PTH promotes bone resorption and raises serum PO_4_^3-^ levels among individuals with healthy renal physiology. Nevertheless, PTH has a dominant role in renal function in excreting PO_4_^3-^, which ultimately reduces its concentration in blood [64]. Another regulator, FGF23, is generated by osteocytes, and osteoblasts are recognized as the primary cause of excessive phosphate excretion in urine and are accountable for regulating PO_4_^3- ^[65]. Raised urinary PO_4_^3-^ elimination and declining phosphate absorption from the gut occur due to reduced calcitriol synthesis by hindering 1α-hydroxylase [66]. FGF23 requires a Klotho cofactor to bind its receptor (FGFR-1c) to exert its bioactivities. Thus, FGF23 is effective only in tissues that can yield Klotho (distal convoluted tubule of the kidneys, parathyroid glands, and the brain). FGF23 and α-Klotho, parathormone, calcitriol, and a number of phosphate transporters in the renal and gastrointestinal systems regulate phosphate homeostasis. Again, genetically persuading hyperphosphatemia origins widespread tissue damage, especially in essential organs [67].

**Figure 2:**
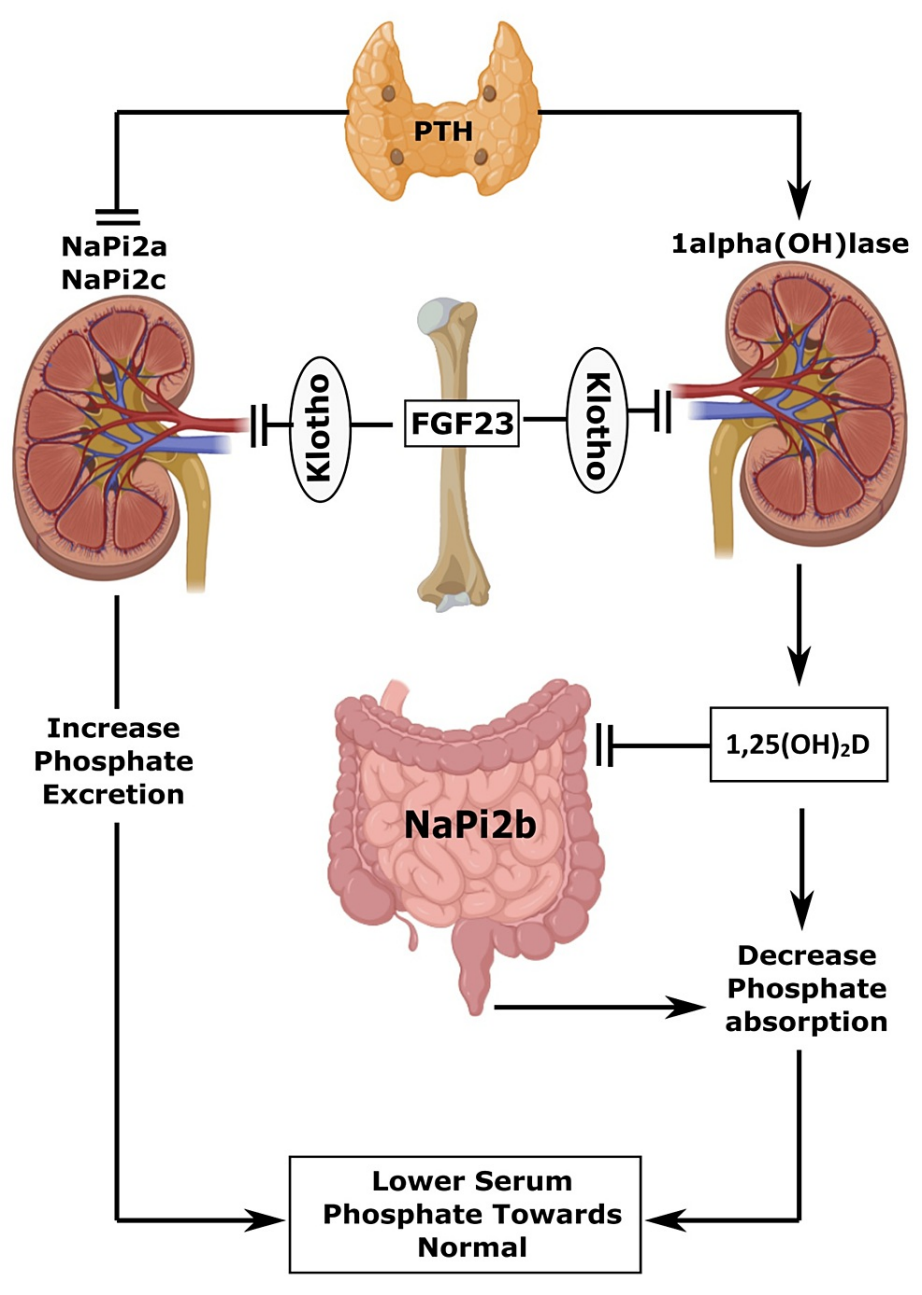
Diagrammatic representation of phosphate balance in humans. PTH: parathyroid hormone; FGF23: fibroblast growth factor-23 Image credit: Susmita Sinha

Researchers found that a transmembrane protein Klotho has various possessions on the physiological control of inorganic electrolytes, principally Ca^2+^ and PO_4_^3-^, and energy biotransformation through the hormonal action of FGF [68]. Klotho is expressed in several organs such as kidneys (distal convoluted tubules), parathyroid glands, liver, brain, and adipose tissue [69]. Among the FGFs, FGF23 can hinder the actions of 1α-hydroxylase and Na/Pi symporter in the kidneys from affecting the general systemic PO_4_^3-^ balance (Figure 2) [70].

The Use of Inorganic Phosphate as a Food Preserver

The addition of Na^+^ salt in food was practiced through ancient times and as early as 5,000-10,000 years [71]. With the advancement of food knowledge, people have started to avoid sodium salt as a food preservative and additive to protect themselves [72]. The increasing severity of hypertension and its prevalence due to high salt-containing food intake in LMICs and high-income countries (HICs) results in the wide application of inorganic PO_4_^3- ^as a foodstuff preserver [73-75]. In the human diet, a significant source of phosphate is foodstuffs originating from milk, fish, and other types of flesh. Approximately 40%-60% phosphate absorption occurs in the gut, which is then carried into the body fluid and distributed across the body, including the renal system [76]. It has been observed that inorganic phosphorus/phosphate is used as preservatives in as high as 100% of factory-processed diets [77]. Factory-processed foods frequently contain food preservatives, often hidden as hazardous toxic elements. In addition, community people and health professionals are unaware of phosphate-containing food preservative-induced toxicity [78]. Excessive phosphate intake results from adding more phosphate to foods and unavailability of hyperphosphatemia-related sufficient information. Previous reports showed that consuming treated foodstuffs (containing inorganic phosphates as preservatives) is associated with several noninfectious illnesses such as T2DM, hypertension, heart muscle dysfunction, obesity, and carcinomas, which are increasing globally [79-81].

Obesity and Insulin Resistance

The modern lifestyle, especially among urbanized in both minimal to average earning and higher earning states, is pervaded by excessive prepackaged food, containing high calories, and is often correlated with low-energy-consuming work [82-85]. High calories with low fiber-containing food and modern jobs that require sitting for a prolonged time that primes to increase weight and fat deposition determine a prevaricate energy equilibrium [86-89]. Thereby, upsetting energy balance frequently disrupts the homeostasis of the liver and adipose tissue. This is expected to be accomplished by instigating the pro-inflammation of immune cells heading toward peripheral insulin resistance (IR) [90-96]. These pathologies ultimately culminate in metabolic syndrome (MetS), represented as multiple noncommunicable diseases, principally abdominal or central obesity, chronically raised blood pressure, dyslipidemia, IR/impaired insulin glucose metabolism, and steatohepatitis [97-99].

Over the last 25 years, multiple studies reported that fatty tissue hyperplasia and hypertrophy frequently correlated with the negative impact of adipose tissue metabolic equilibrium [100,101]. Additionally, various studies said that tumor necrosis factor-alpha (TNF-α) was significantly raised among obese individuals, eventually rooted in IR, and surfaced the notion of an inflammatory element of obesity [86,102-105]. This inflammatory pathological issue is known as “meta-inflammation,” a clinical disease of chronic, low-grade inflammation noticed among obese patients [106,107]. This subthreshold inflammation, mainly observed in white adipose tissue (WAT), causes regular initiation of the innate immune system and leads to IR, impaired glucose tolerance (IGT), and T2DM [108-110]. WAT as lipids is the biological location of energy storage and is freshly documented for several important physiopathological activities [111-115]. Multiple studies reported that the WAT of overweight individuals is frequently penetrated by macrophages, which may act as a focal basis for the localized formation of pro-inflammatory cytokines [107,116-118]. WAT has generated a diverse spectrum of inflammatory particles, including TNF-α and interleukin-6 (IL-6), negatively impacted locally and systematically [110,119,120]. These pro-inflammatory cytokines originate IR and T2DM in fatty tissue, striated muscle, and the liver by impeding the insulin signal system, causing defective insulin signaling and glucose uptake [121-123]. Furthermore, cytokines (e.g., adipokines, hepatokines, inflammatory cytokines, myokines, and osteokines) also subsidize anomalous lipid metabolism [124-126].

Leptin is one of the adipokines produced by adipocytes; increasing adipose tissue mass elevates leptin concentration in plasma [127]. Again, IL-6 hinders the expression of insulin receptors and adiponectin. A recent study suggested that TNF-α is concerned with the inflammatory reactions that link centripetal adiposity by means of IR and inhibit the insulin-stimulated tyrosine kinase activity of the insulin receptor and substrate-1 (IRS-1), consequently decreasing insulin-dependent glucose transporter (GLUT-4) in the cells [128]. TNF-α also activates sphingomyelinase, thus stimulating ceramide production and accumulation in adipose tissue, inhibiting adiponectin secretion [129]. Hormone-sensitive lipase (HSL) acts as an intracellular neutral lipase that hydrolyzes several lipid esters, such as hydrolyzing diacylglycerol (DAG) into monoacylglycerol (MAG) or entirely with the let-out free fatty acid (FFA). The insulin is switched on principally by β-adrenergic catalyst force and rendered inoperative. However, it requires phosphorylation and translocation into lipid driblet to clarify its pursuit. Thus, the discharge of fatty acids is restricted in raised glucose concentrations [130], as insulin is unable to hinder HSL in an insulin-resistant state, causing an elevated FFA accumulation [131]. Additionally, outlying tissues started utilizing FFAs in β-oxidation as a power generation process or a substratum to produce additional lipids. Further lipid deposition in skeletal muscle and the liver accounts for the initiation of IR [132]. In T2DM, insulin sensitivity is declining. Throughout the aforementioned condition, the hormone becomes futile and is primarily opposed by hyperinsulinemia to continue glucose equanimity. Ultimately, insulin manufacturing declines progressively, and T2DM occurs. Researchers found that T2DM patients have more BMI with a higher body fat percentage, and increased adiposity in obese individuals tends to develop metabolic syndrome, T2DM, and cardiac morbidities [133]. This augmented adipose tissue mass encourages IR through many inflammatory processes, amplified FFA discharge, and adipokine dysregulation, resulting in T2DM.

A study showed that ingesting prepackaged edibles increases foodstuff-related chronic illnesses such as overweight, obesity, T2DM, and hypertension [134]. Excess weight and obesity result from additional consumption of sugar-sweetened beverages containing high calories and high phosphates [135]. Food additives and preservatives improve the taste, and the desire to take processed foodstuffs is greater, resulting in obesity [136]. Again, highly flavorful processed edibles stimulate the brain’s reward and motivation pathways, raising the possibility of seeking processed food and its consumption [137]. The peroxisome proliferator-activated receptor (PPAR) family is related to the energy-yielding metabolic process. The nuclear receptors PPARα, PPARδ, and PPARγ act as lipid sensors regulating the expression of several genes crucial for controlling energy metabolism [138]. PPARγ increased Klotho expression during the early period of adipocyte differentiation. α-Klotho is a humoral factor that overpowers growth factor receptors such as insulin and insulin-like growth factor-1 (IGF-1) [139]. In addition, fetuin-A, a blood serum glycoprotein generated by the liver, can attach to the β subunit of the insulin receptor, jamming the metabolic arm of insulin action, thus hindering insulin-dependent activation of the receptor. This disruption of fetuin-A mediated insulin receptor control in defective glucose disposal, IR, and fatty liver [140].

Food Additive and Preservative Adversaries on Vascular Physiology

Factory-made food contains added sugar and fat and holds several additional molecules to rise deliciousness, adjust consistency, and extend durability. These supplementary goods are together termed foodstuff additives [141]. Foodstuff additives must comply with rigorous safety tests to determine their possible adverse impacts on human health, following national and international guiding principles [142,143]. Vascular pathology is often instigated using dyslipidemia and hyperphosphatemia [144-146]. Statins have been reported globally for their effective management of dyslipidemia [147-149], whereas there are still no appropriate therapeutic options for treating hyperphosphatemia [150,151].

In contrast, tangential colloidal nanoparticles consisting of crystalized fetuin-A attached with Ca_3_(PO_4_)_2_ in the blood had been notified to be the originator of vascular injury and the formation of solidified clumps in soft tissues [152]. Hyperphosphatemia has been related to death, cardiac events, and vascular ossification in human studies for the last few years [153]. Adverse outcomes from increased phosphate are incompletely understood; until now, evidence recommends an immediate influence of PO₄³⁻ on vascular ossification and FGF23 and calcitriol transition.

Inorganic phosphate has numerous physiological functions, for instance, skeletal development, bone mineralization, adenosine triphosphate (ATP) generation, plasma membrane probity conversion, cell signaling (control of subcellular procedures over protein phosphorylation of crucial enzymes), signal transduction, nucleotide metabolism, enzyme control system by nucleic acids, acid-base equilibrium preservation from end to end protecting effect in urine, and platelet accumulation [154]. Again, inorganic phosphate is mainly needed to keep up essential functions and is frequently obtained from foodstuffs containing organic phosphorus. Foodstuff preservers having inorganic phosphate are absorbed solely and readily (80%-100%) from our intestines [155]. Researchers revealed that persistent intake of mineral phosphate started accumulating in the different body systems, and high plasma concentration of phosphate can cause a mess with the hormonal regulation to maintain balanced phosphate echelons in physically fit people [156].

Phosphate and Mitochondrial Dysfunction

Mitochondria are membranous organelles that produce ATP by transforming glucose to provide cell nourishment [157]. Inside the mitochondrion of β-cells of islets of Langerhans of the pancreas, ATP formation controls insulin secretion, and membranous transportation of inorganic phosphate for ATP formation ensues by type III Na^+^-dependent cotransporters PiT1/2 [158]. In vitro experimentations on cultured β-cell (INS-IE) and pancreatic islet cells have revealed that concentrations of inorganic phosphate outside the cells (1-5 mM) prompted mitochondrial malfunction; in addition to this, it was observed that depressed ATP synthesis and rise of ROS related to low insulin content decreased insulin release and increased endoplasmic reticulum (ER) stress [159]. Investigators found that the collection of higher concentration of Ca_3_(PO_4_)_2_ inside the mitochondrion is similarly connected to the entrance of the mitochondrial permeability transition pore (mPTP), a protein complex involving the mitochondrial membranes, and space allows substances equal to 1500 Dalton size to pass in and leave the mitochondrial milieu [160]. This invasion can cause distension and disruption of the external mitochondrion membrane and discharge mitochondrial proapoptotic features, subsequent in necrotic or apoptotic cell death [161]. Again, free Ca^2+^ hitched higher inorganic phosphate content and excessive calcium-phosphate product formation in serum [162].

Vascular Calcification

**Figure 3:**
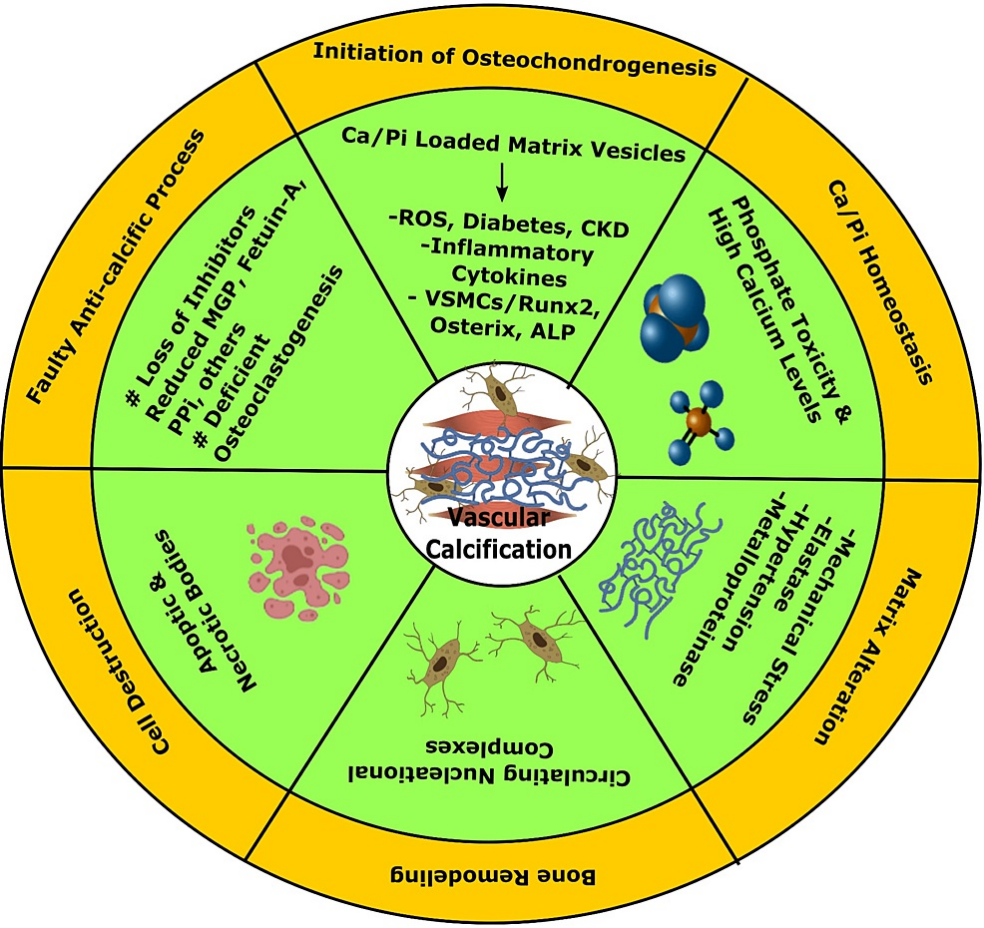
Mechanisms for vascular calcification. (1) Faulty anti-calcific process due to lack of expressed mineralization inhibitors, (2) product of vesicular matrices act as a nest for Ca_3_(PO_4_)_2_ deposition initiating the osteochondrogenic process, (3) necrotic debris from cellular death by apoptosis perform as nucleation of apatite, (4) irregular Ca/Pi balance sediments Ca_3_(PO_4_)_2_ hydroxyapatite, (5) nucleational complexes help deranged mineralization, and (6) matrix alterations by biodegradable irritants are also engaged in vascular calcification. MGP: matrix Gla protein; PPi: inorganic pyrophosphate; ROS: reactive oxygen species; CKD: chronic kidney disease; VSMCs: vascular smooth muscle cells; ALP: alkaline phosphatase Image credit: Susmita Sinha

Vascular smooth muscle cells (VSMCs) are essential in the pathology of vascular ossification. VSMCs may turn into bone-forming and cartilage-forming cells in the progression of stress. Osteoblast-like cells that comprehend hydroxyapatite crystals look as if in the extracellular matrix in the course of vascular smooth muscle ossification (Figure 3) [163].

**Figure 4:**
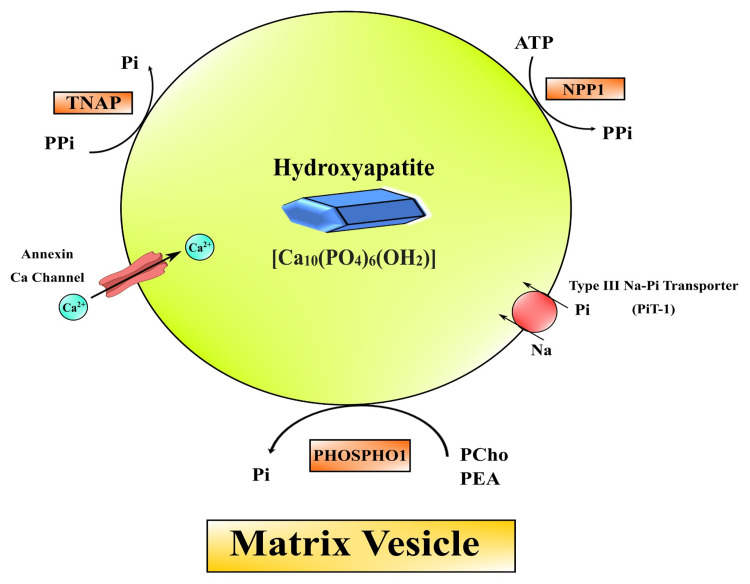
Formation of hydroxyapatite crystal in matrix vesicle. TNAP: tissue-nonspecific alkaline phosphatase; NPP1: nucleotide pyrophosphatase/phosphodiesterase; PHOSPHO1: phosphoethanolamine/phosphocholine phosphatase 1; PEA: phosphoethanolamine; PCho: phosphocholine Image credit: Susmita Sinha

Vessel ossification is of two types: one is intimal calcification, and the other is medial calcification. Intimal calcification may be responsible for atherosclerosis and chronic inflammation, and VSMCs are converted to bone-forming cells. In addition, medial layer ossification might cause elastin degradation, extracellular matrix remodeling events, and increased phosphate in the blood, resulting in chronic kidney disease (CKD), hypertension, and T2DM [164]. The factors involved in facilitating and suppressing vessel ossification are listed in Table 1 [165]. The physiological calcification process begins with accumulating the cell derivative matrix vesicles into the extracellular matrix. Hydroxyapatite crystal deposits are observed in vivo in a human being, a vascular cell model (Figure 4) [166].

**Table 1:** Chief facilitator and suppressor elements concerned in vessel ossification. BMP: bone morphogenetic protein; MGP: matrix Gla protein; MMP: matrix metalloproteinase; BMP7: bone morphogenetic protein 7; Runx2: runt-related transcription factor 2; PTH: parathyroid hormone; RANKL: receptor activator of nuclear factor-kappa B ligand; SOX9: sex-determining region Y-box 9

Facilitators	Suppressors
BMP2, BMP4, and BMP6	MGP
Osteocalcin	Osteopontin
Alkaline phosphatase (SOX9)	Osteoprotegerin
	Vitamin K
Osterix	Magnesium
MMP2, MMP3, and MMP7	BMP7
Runx2	Fetuin-A
Ca^2+^	Klotho
PO_4_^3-^	PTH
C_6_H_12_O_6_	Pyrophosphate
Advanced glycation end products	Carbonic anhydrase
Oxidized low-density lipoproteins	
Collagen I	
RANKL	

Phosphate Toxicity and Vascular Impediment

The progression of the calcification procedure in vessels by persuading the formation of cells similar to bone-forming cells from VSMCs can result from hyperphosphatemia [167]. Elevated PO_4_^3-^ upregulates the expression of PiT in VSMCs [168]. Investigational studies specified that the activation of GK1/nuclear factor-kappa B (NF-κB), Wnt/β-catenin signaling, and upregulation of osteogenesis transcript factors Runx2, msh homeobox 2 (MSX2), sex-determining region Y-box 9 (SOX9), and osterix are usual processes of osteogenesis distinction of VSMCs, among which runt-related transcription factor 2 (Runx2) has an important part [169]. Again, cytokines that tend to cause inflammation are a group of endogenic peptides primarily formed by immune cells, as well as interleukin (IL)-1β, TNF-α, and IL-6. In vitro studies showed that IL-1β and TNF-α could excite the in-cell nuclear factor-kappa B (NF-κB) signaling, and IL-6 activates the BMP-2Wnt/β-catenin pathway to encourage the ossification of VSCMs [170]. This research has discovered that growth arrest-specific transcript 5 (GAS5)/miR-26-5p/phosphatase and tensin homolog (PTEN), FGFR1c-MEK/ERK-GALNT3 path, and alpha Klotho deficiency are important processes of vessel ossification. In addition to this, huge PO_4_^3-^ inducement downregulated the expressiveness of a long-chain noncoding RNA (lncRNA), growth-specific inhibitor, and growth arrest-specific transcript 5 (GAS5) in a person’s aortic non-striated muscle cells, which deteriorated the hindrance of small nucleolar RNA miR-26b-5p and thus its subsequent focus protein phosphatase and tensin homolog (PTEN). PTEN is a protein/lipid phosphatase that encourages mesenchymal cells’ osteogenesis by augmenting the expressiveness of runt-related transcription factor 2 (Runx2) [171,172]. Another study predicate that more PO_4_^3-^ bound to FGFR1c actuates the down-streaming of extracellular regulated protein kinase mitogen-activated protein kinase kinase (MEK)/extracellular signal-regulated kinase (ERK) phosphorylation and encourages the expressiveness of osteogenesis-associated proteins, such as BMP-2 and BMP-7 [173]. In high phosphate concentration, VSMCs process and push pyrophosphate extracellularly. Again, fetuin-A is a protein that can attach directly to Ca2+ or hydroxyapatite to hinder the progress of hydroxyapatite crystals [174]. In addition, tissue-nonspecific alkaline phosphatase (ALP) is amplified, resulting from the repress production of calcification inhibitions by high phosphate levels. ALP causes the deposition of microcalcification and the development of calcium phosphate crystals [175]. An extracellular matrix renovation in the tunica media occurs in response to phosphate toxicity. Accelerated generation of matrix metalloproteinases (MMPs) by VSMCs, such as MMP2 or MMP9, and additional deterioration of quite a few extracellular matrix proteins and elastin filaments make an available extra den for calcium phosphate precipitation [176-178].

A disproportionate quantity of the cysteine protease cathepsin S escorts to the breakup of elastin and the production of bioactive elastin peptides, which can perform straightly on VSMCs to hasten vascular calcification in phosphate excess [179]. Moreover, osteogenic/chondrogenic transdifferentiation of VSMCs induced by PO_4_^3-^ misplace the contractile constitution and acquire the same characteristics as osteoblasts and chondroblasts and exhibit osteogenic transcription factors, e.g., msh homeobox 2 (MSX2), core-binding factor α-1 (CBFA1) or osterix, and chondrogenic transcription factor with sex-determining region Y-box 9 (SOX9) [180].

Phosphate Toxicity and Deranged Vascular Calcification in Obesity

Phosphate toxicity is a notable consequence of impaired renal function. However, the pathological outcomes of hyperphosphatemia in obese individuals have no longer been explored in equal intensity (Table 2). Investigators found that the prevalence of obesity-associated renal impairment is rising worldwide; along with this, experiments showed vast soft tissue and vessel ossification in the renal system, great vessels, lungs, and other organs in genetically modified hyperphosphatemic obese experimental animals [181]. Phosphate is an important constituent of our everyday foodstuffs and is primarily acquired from proteins. Over the last few years, PO_4_^3-^ has been abundantly utilized as an artificial food enhancer in treated edibles, carbonated drinks, cola, fast foods, precooked packaged foods, and processed meats [182]. Consuming high phosphate-containing foods in excess may cause various noncommunicable diseases, principally abdominal or central obesity (Table 2), chronically raised blood pressure, dyslipidemia, IR/impaired insulin glucose metabolism, and steatohepatitis.

FGF23 is derived from osteocytes, and FGF23 is capable of reducing phosphate levels to normal by two processes. The first process is by lowering renal phosphate reabsorption by suppressing type II Na^+^-PO_4_^3-^ symporters (NaPi-2a and NaPi-2c) in the proximal convoluted tubule and inhibiting the obstruction of 1-alpha-hydroxylase. The subsequent process involves the upregulation of 24-hydroxylases, which reduces 1,25-dihydroxycholecalciferol concentration, promotes intestinal phosphate uptake, and thus lowers serum PO_4_^3-^ concentration [183]. Researchers showed a strong relation linking high FGF23 and hypertrophic cardiac myopathy in obese young people without T2DM [184]. In another study, researchers evaluated that the levels of FGF23, alpha Klotho, and 1-25(OH)2D3 were remarkably reduced in obese subjects than in control [185].

Phosphate Toxicity, T2DM, and Impaired Vascular Function

More than phosphate toxicity, the Pi level plays an integral part in the progression of calcified vessels in T2DM [186]. Ca_3_(PO_4_)_2_ deposits as hydroxyapatite, predominantly present in DM and CKD, resulting from plaque formations in tunica media and intima of the blood vessels [187]. Researchers observed that human VSMCs incubated in high Ca^2+^ and PO_4_^3-^ concentrations instigated impairment to VSMC mitochondria, lessened mitochondrion action, augmented superoxide creation, and caused calcified vessels through altering VSMC metabolic processes [188]. The ossification of cultivated VSMCs persuaded under elevated PO_4_^3-^ conditions was related to the augmented inflammatory cytokine expressiveness of IL-1β, IL-6, and TNF-α, along with phosphorylation and triggering of the toll-like receptor-4 (TLR-4)/nuclear factor-kappa B (NF-κB) signaling pathway that is similarly linked to long-lasting inflammation [189].

**Figure 5:**
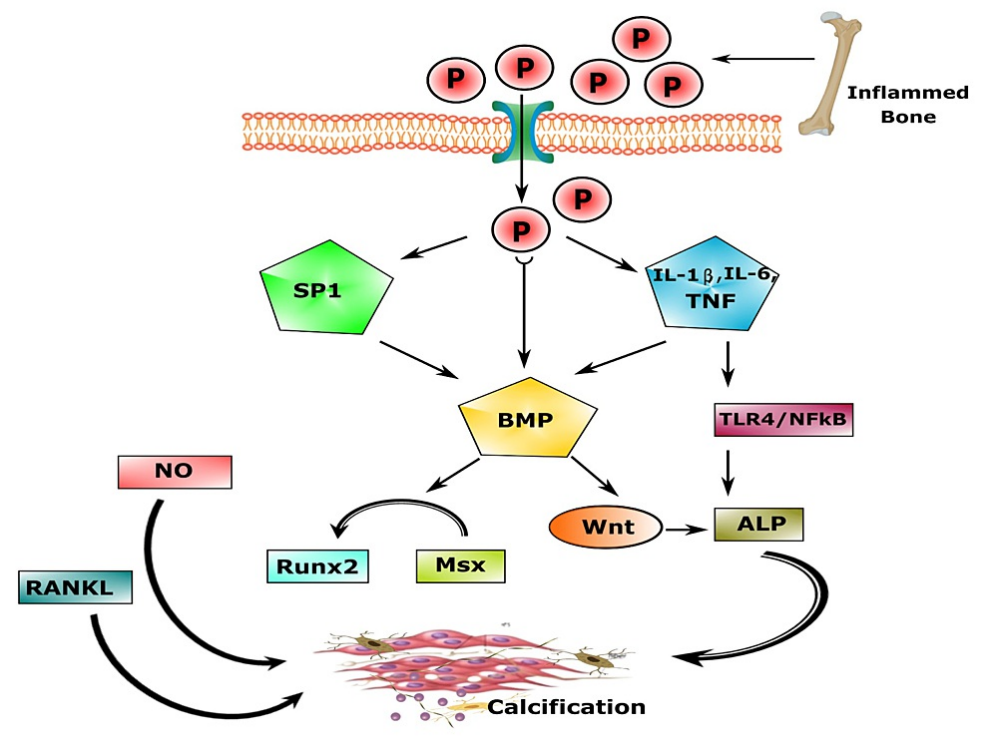
Mechanism of the formation of vascular calcification in T2DM due to phosphate toxicity. TNF: tumor necrosis factor; IL: interleukins; BMP: bone morphogenic protein; Runx2: runt-related transcription factor 2; NF-κB: nuclear factor-kappa B; MSX: msh homeobox; ALP: alkaline phosphatase; RANKL: receptor activator of nuclear factor-kappa B ligand Image credit: Susmita Sinha

Modification in bone metabolism leads to raised serum phosphate in T2DM, especially in CKD patients, and serum concentrations of osteocalcin decline in diabetic patients [190]. Researchers showed that serum alkaline phosphatase (ALP) action is upregulated in diabetic subjects, and ALP disintegrates inorganic pyrophosphate (PPi) [191]. Phosphate toxicity also represses the distinction of monocytes/macrophages into an osteoclast-like cell in vitro by downregulated RANKL-driven signaling. In contrast, diabetes-associated oxidative stress has a prohibitory influence on the segregation of osteoblasts and bone marrow cells (BMCs) [192]. Raised extracellular phosphate concentrations to enhance phosphate influx into VSMCs, initiating Runx2 and osteocalcin (Figure 5) [193]. Atherosclerotic vascular calcification was also encouraged throughout the phosphatidylinositol 3-kinase (PI3K)/Akt (protein kinase B) signaling pathway in trial animals with DM via Runx2 upregulating the transdifferentiation of VSMC into similar bone-forming cells [194]. In addition, when foods rich in PO_4_^3-^ are supplied to experimental animals, the phosphatidylinositol 3-kinase/Akt signaling pathway is initiated [195].

Elevated serum PO_4_^3-^ concentrations cause liberation of FGF23 from bone to decrease serum PO_4_^3-^ by reducing renal PO_4_^3-^ reabsorption and rising urinary phosphate excretion [196]. Phosphate toxicity results in long-lasting renal disorder and end-stage renal disease and is also linked to diabetic nephropathy. Kidneys have abundant mitochondria in the proximal convoluted tubule; defects in mitochondria may cause reduced ATP synthesis and renal malfunction in diabetic kidney illness. Again, renal proximal convoluted tubular cells are found to be infiltrated with calcium phosphate precipitates after culturing in osteogenic environments [197].

Another experimental study showed that phosphate toxicity results in uremia in trial rats, and sediments of calcium phosphate were found in the basal ganglia. Furthermore, excess dietary intake of phosphate instigated malnutrition and inflammation, including vascular calcification in experimental rodents with uremia [198]. Lower vitamin D levels are frequently observed in T2DM, linked to pain in diabetic peripheral neuropathy, but the relationship is not established yet [199]. Phosphate toxicity causes decreased bioactive vitamin D synthesis and reduced intestinal absorption of PO_4_^3-^ and Ca^2+^.^ ^Furthermore, it decreases renal Ca^2+^ and PO_4_^3- ^reabsorption and causes impaired bone mineralization, thereby reducing serum PO_4_^3-^ levels. This could be an intervening reason to clarify the relationship of reduced vitamin D echelons in DM subjects with hurting diabetic peripheral neuropathy [200]. A recent study found that calcium phosphate deposition in mitochondria caused neuronal mitochondria defect and neurodegeneration [201].

Phosphate Toxicity Affecting Renal Physiology

There is a balance in PO_4_^3-^ concentration maintained by the kidneys during physiological conditions. After filtration, PO_4_^3-^ is taken up in the renal tubular epithelium via Na^+^-Pi symporters [202]. Kidney bone derangements involve renal insufficiency, osteoporosis, and vascular calcification resulting from phosphate toxicity. Excessive phosphate consumption through foodstuffs causes disturbance in renal filtration, increasing phosphate retention and thus raising phosphate levels [203]. Renal proximal tubular epithelial cells accommodate three categories of Na/Pi symporters. The type 2 symporter group includes Na/Pi2a, and Napi2c are considered as much dynamic PO_4_^3-^ taken up in the kidney. Unlike the type 2 symporter, the type 1 symporter (NPT1) is primarily an anion carrier.

In contrast, the type 3 symporter system comprises two transporters, PiT1 and PiT2, and their role in PO_4_^3-^ transportation is a sphere of study [204]. In addition, alpha Klotho, the cofactor for FGF23 bioactivities, is available mainly in the distal tubule [205]; in addition to this, CKD patients show decreased levels of alpha Klotho, which causes FGF23 to be inoperative, thus limiting the kidneys’ ability to eliminate phosphate [206].

**Figure 6:**
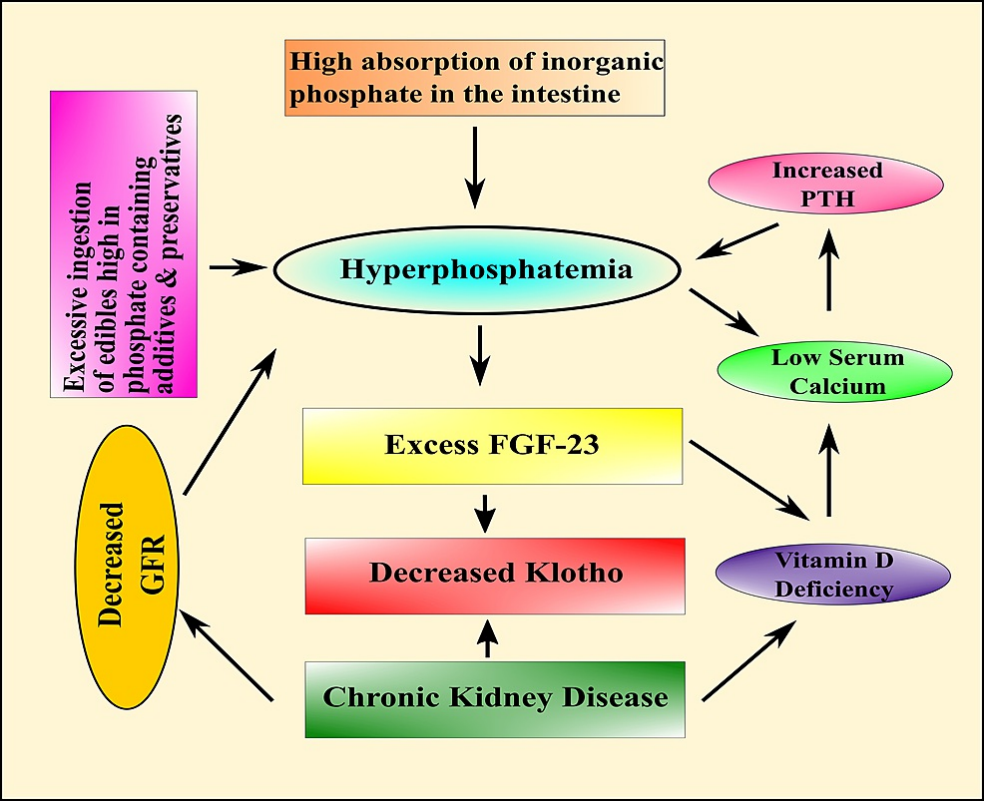
Schematic presentation of the effects of phosphate toxicity on the renal system. PTH: parathyroid hormone; FGF23: fibroblast growth factor-23; GFR: growth factor receptor Image credit: Susmita Sinha

Another in vivo study showed that phosphate toxicity in alpha Klotho-ablated mice accompanies the increased renal activity of Na/Pi2a; ensuing phosphate toxicity (Figure 6) begins from an unbalanced accumulation of PO_4_^3-^ in the body [207]. Bone health and overall existence can be altered in the presence of more than normal PO_4_^3- ^levels related to foodstuffs; it can contribute to the progression of the human aging process [208]. Calciprotein particles (CPPs) are identified in the bloodstream, particularly in CKD patients, as a protagonist in phosphate-arbitrated renal tissue grievances [209]. The estimated prevalence of CKD worldwide is 8%-16% [210]. Edible PO_4_^3-^ excess may cause an increased PO_4_^3-^, a burden that results in renal tubular injury and interstitial fibrosis. There is a raised level of the possibility of evolving poor renal function with high serum phosphate [211]. Recent studies showed that treating human embryonic kidney cells (HEK-293) with increased concentrations of Pi for a short while persuades cytotoxicity. The investigators also found that phosphate in a dose-dependent phenomenon affects the cell division cycle and cell survival. In HEK-293 cells, an increase in Pi caused a notable rise in Akt phosphorylation, and a gradual rise in Pi concentrations caused an additional increase in Akt phosphorylation. Thus, Pi enhances Akt stimulation, resulting in cell cycle progression encouraged by the PI3K/Akt pathway. As processed foodstuffs (Table 2) and drinks contain high phosphate concentrations, CKD patients should cut their consumption of these food products [212,213]. Multiple studies reported that high levels of phosphate preservatives are found in convenience food or ready-to-eat meal [77,214-229]. Additionally, high serum phosphorus is incorporated with the cardiac death toll in long-lasting kidney diseases [78,230-232].

**Table 2:** Depicting processed food and its implication for obesity, renal impairment, and endocrine issues. HDL: high-density lipoprotein; IR: insulin resistance; T2DM: type 2 diabetes mellitus

Reference	Country involved	Study population	Study design	Results
Anyanwu et al. [214]	Indonesia	N = 31,160	Cross-sectional study	The prevalence of overweight and obesity among the study population was 30.9% and 17.4%, respectively. Younger age and male gender significantly correlated with higher eating habits of processed foods.
Almandoz et al. [215]	USA	N = 404	Cross-sectional study	Around 5% of the study population put on additional weight. These individuals were found to have more stressful life patterns, anxiety, and depression and were often less deprived of poor physical activity. They preferred to take less healthy meals such as more processed, ready-to-eat foods. They also frequently overindulge with binge eating disorder (BED).
Rey-García et al. [216]	Spain	N = 1,312	Prospective cohort study	The consumption of processed food was statistically significantly (p = 0.026) correlated with declining renal physiology. Additionally, high consumption of ready-to-eat meals is sovereignly related to more than 50% possibility of jeopardizing renal physiology among the Spanish elderly community.
Sandoval-Insausti et al. [217]	Spain	N = 3,521	Prospective cohort study	Higher convenience store food (fast food) ingestion was linked with central obesity among Spanish individuals aged 60 and over.
Beslay et al. [218]	France	N = 110,260	Prospective study	French people consuming more ultra-processed food were positively related to weight gain in BMI and a greater risk of overweight and obesity.
Donat-Vargas et al. [219]	Spain	N = 1,082	Prospective cohort study	Individuals who were principally dependent on ready-to-eat meals are frequently related to hypertriglyceridemia or low HDL cholesterol.
Llavero-Valero et al. [220]	Spain	N = 20,060	Prospective cohort study	This study reported that those people who chiefly depend on processed food are independently associated with a higher risk for type 2 diabetes mellitus.
Montero-Salazar et al. [221]	Spain	N = 1,876	Cross-sectional study	Among middle-aged persons, consuming processed food daily was related to coronary atherosclerosis. Additionally, it has been observed that the risk of cardiovascular issues doubles with consuming 500 g daily than 100 g daily.
Du et al. [222]	USA	N = 13,548	Cross-sectional study	Greater ultra-processed food consumption was related to a higher risk of coronary artery disease among US adults who are 45-65 years. Additionally, it has been reported that linear relation was observed between packaged food consumption and the hazard of coronary artery disease.
Kurniawan et al. [223]	Taiwan	N = 41,128	Cross-sectional study	Multivariate linear regression analysis revealed that high consumption of packed food, especially flesh and rice/flour products, and low intake of plant origin foods, e.g., fruit and dark-colored vegetables, frequently increases the possibility of cardiovascular disease and weakened renal function among individuals aged 40-95 years.
Kurniawan et al. [224]	Taiwan	N = 25,569	Prospective study	This study revealed that eating habits were related to metabolic syndrome.
Vogelzangs et al. [225]	The Netherlands	N = 634	Prospective cohort study	It has been reported that Dutch nondiabetic obese individuals possess both liver and muscle IR with high valine, isoleucine, oxoisovaleric acid, alanine, lactate, and triglycerides and lower levels of glycine. Additionally, the hepatic IR index was statistically significant and associated with amplified levels of leucine, hydroxyisobutyrate, tyrosine, proline, creatine, and n-acetyl and lower levels of acetoacetate and 3-OH-butyrate.
Wang et al. [226]	China	N = 94,952	Prospective cohort study	IR had a robust relationship with the Chinese obese community.
Niu et al. [227]	China	N = 369	Retrospective study	It has been reported that obesity remains the principal instigating factor for several noncommunicable disorders. These include IR, T2DM, hypertension, hyperuricemia, and metabolic syndrome.
Martins et al. [228]	Brazil	N = 153	Cross-sectional study	This study revealed that the elderly community under hemodialysis frequently has an unhealthy diet than healthy elderly individuals. Additionally, it has been further spotted that these dialysis patients consume relatively worse processed food on non-dialysis days.
Watanabe et al. [44]	Brazil	N = 100	Cross-sectional descriptive-analytical study	This study revealed that protein with phosphate-containing preservatives in prepackaged food was significantly higher (p < 0.0001) than in fresh foods, as well as the phosphate-to-protein ratio.

Professional annotation

Excessive, constant consumption of foods containing high phosphate levels has been linked to the amplified incidence of several long-term disorders and metabolic disorganization. Again, oxidative stress has a crucial role as an important influence relating obesity with its allied complexities. Obesity alone can persuade systemic oxidative stress via numerous biochemical processes, for instance, superoxide generation from nicotinamide adenine dinucleotide phosphate (NADPH) oxidases, oxidative phosphorylation, glyceraldehyde auto-oxidation, protein kinase C activation, and hexosamine pathways [233]. Oxidative stress originated from reactive oxygen species (ROS), which adipokines can provoke yielded from adipocytes in obesity. Oxidative stress in obesity involves multiple processes, namely, mitochondrial and peroxisomal oxidation of fatty acids and excessive use of oxygen [234]. Obesity enhances oxidative stress and exhibits inflammation linked with insulin resistance and endothelial affliction [235]. Endothelial dysfunction is expressed as decreased biological availability of vasodilators such as nitric oxide (NO) and raised endothelium-derived contractile factors [236]. Inorganic phosphates (Pi) assimilate into the mitochondria, producing ROS, leading to ER stress and mitochondrial malfunction through the opening of the mitochondrial permeability transition pore (mPT). Increased blood glucose levels raised the requirement for insulin, which put a considerable load on the endoplasmic reticulum (ER). Proinsulin translation in response to increased blood glucose levels provokes extreme protein folding in ER, leading to ER stress. During ER stress, few β-cells pass away, and the remainder of β-cells intensify ER workload. In addition, if ER stress continues, β-cell numbers decrease, resulting in DM [237].

Furthermore, phosphate toxicity may result in inorganic phosphate excess instigating bone-producing cell distinction and vascular smooth muscle ossification facilitated by oxidative stress. For this reason, insulin-liberating cells are immensely susceptible to oxidation, and phosphate toxicity might be incredibly destructive to the pancreatic β-cells [238]. A study showed that ingesting prepackaged edibles increases foodstuff-related excess weight and obesity resulting from additional consumption of sugar-sweetened beverages containing high calories and high phosphates [239]. Moreover, researchers found that FGF23, vitamin D, and PTH mainly facilitate the endocrine control of phosphate balance. Disturbance of harmony among these factors, either solo or in alliance, may provoke phosphorus disproportion, and the inhibition of the FGF23 Klotho system can cause hyperphosphatemia with widespread tissue injury instigated by hyperphosphatemia [240]. Besides, researchers showed that without Klotho, elevated levels of FGF23 cannot control systemic phosphate homeostasis [241]. Hyperphosphatemia is one of the leading causes of decreased renal function [242]. In addition, another study acquired leptin-deficient obese experimental animals to see the effect of phosphate toxicity on obesity and discovered a wide range of nonskeletal tissue and calcified vessels in the kidneys, aorta, lungs, and other organs of these obese mice [243]. In particular, the biological activity of FGF23 depends upon alpha Klotho, available principally in the distal convoluted tubule. CKD patients showed diminished alpha Klotho levels, resulting in restricted urinary phosphate excretion. Phosphate toxicity results in chronic kidney and end-stage renal disease and is also linked to diabetic nephropathy [244]. Thus, diet-induced phosphate burden could impair kidney functions through tubular injury and interstitial fibrosis, increasing the risk of kidney disease.

## Conclusions

Collecting proof recommends that dietary elements, for instance, foodstuff flavorings and preservatives, contribute to the genesis of obesity and T2DM. With further research, the clinical intervention to reduce phosphate consumption and subsequent hyperphosphatemia has an enormous capability for upcoming advancements in the management and progression of T2DM and associated complications. There exist different types of biomolecular processes that have crucial influences on the pathophysiology of the ossification of blood vessels. In addition, conservative and alternate prevention processes, for example, preserving a well-balanced eating regimen and remedial strategies, are prospects to reduce the vascular calcification problem in T2DM due to hyperphosphatemia. It is becoming further noticeable from an observational study and human trials that phosphate toxicity characteristics may arise after consuming foodstuffs rich in PO_4_^3-^. Recent survey results highlight that people are unaware of the unseen supply of PO_4_^3-^ in their food and emphasize the necessity for awareness-raising activities of the hazard produced through foods with veiled PO_4_^3-^ constituents.

There are no convincing epidemiological connections between food additives and preservatives, obesity, and T2DM; clinical trials are a time-demanding issue for establishing an association. In addition, extensive and random testing with challenging endpoints is instantly needed. To lessen the PO_4_^3-^ problem, alertness of its nutritional origin is crucial. Suitable dietary instruction might improve phosphate control and help the whole community with regular renal performance. Information about PO_4_^3-^ additives, PO_4_^3- ^bioavailability, and the phosphate-to-protein ratio would be beneficial data for creating intervention strategies. Food-associated hyperphosphatemia is seemly an emerging international health concern. Without measures to modify ultimate dietary consumption, it will probably exert damaging outcomes on fitness and ailments. Future studies should look into alternate eating regimen concepts with lower glycemic index and safe levels of PO_4_^3-^ quantity.

The following are the highlights of the present article. First, ready-to-eat food consumption is associated with obesity and several noninfectious illnesses such as cardiovascular diseases, hypertension, diabetes mellitus, and tumors. Second, insulin-secreting cells are extremely susceptible to oxidative stress, and phosphate toxicity might be incredibly injurious to pancreatic β-cells. Third, atherosclerotic vascular calcification was also encouraged throughout the phosphatidylinositol 3-kinase (PI3K)/Akt (protein kinase B) signaling pathway in trial animals with DM via Runx2, which upregulated the transdifferentiation of VSMCs into osteoblast-like cells. Lastly, phosphate toxicity results in chronic kidney diseases and end-stage renal disease and is also linked to diabetic nephropathy.

## References

[1] Poti JM, Braga B, Qin B (2017). Ultra-processed food intake and obesity: what really matters for health-processing or nutrient content?. Curr Obes Rep.

[2] Costa CS, Rauber F, Leffa PS, Sangalli CN, Campagnolo PD, Vitolo MR (2019). Ultra-processed food consumption and its effects on anthropometric and glucose profile: a longitudinal study during childhood. Nutr Metab Cardiovasc Dis.

[3] Hales CM, Fryar CD, Carroll MD, Freedman DS, Aoki Y, Ogden CL (2018). Differences in obesity prevalence by demographic characteristics and urbanization level among adults in the United States, 2013-2016. JAMA.

[4] Myers CA, Mire EF, Katzmarzyk PT (2020). Trends in adiposity and food insecurity among US adults. JAMA Netw Open.

[5] Flegal KM, Carroll MD, Kit BK, Ogden CL (2012). Prevalence of obesity and trends in the distribution of body mass index among US adults, 1999-2010. JAMA.

[6] Hruby A, Hu FB (2015). The epidemiology of obesity: a big picture. Pharmacoeconomics.

[7] Mohammadbeigi A, Asgarian A, Moshir E, Heidari H, Afrashteh S, Khazaei S, Ansari H (2018). Fast food consumption and overweight/obesity prevalence in students and its association with general and abdominal obesity. J Prev Med Hyg.

[8] Bhurosy T, Jeewon R (2014). Overweight and obesity epidemic in developing countries: a problem with diet, physical activity, or socioeconomic status?. ScientificWorldJournal.

[9] De Silva AP, De Silva SH, Haniffa R (2015). A cross sectional survey on social, cultural and economic determinants of obesity in a low middle income setting. Int J Equity Health.

[10] Li L, Sun N, Zhang L (2020). Fast food consumption among young adolescents aged 12-15 years in 54 low- and middle-income countries. Glob Health Action.

[11] De Vogli R, Kouvonen A, Gimeno D (2014). The influence of market deregulation on fast food consumption and body mass index: a cross-national time series analysis. Bull World Health Organ.

[12] Maguire ER, Burgoine T, Monsivais P (2015). Area deprivation and the food environment over time: a repeated cross-sectional study on takeaway outlet density and supermarket presence in Norfolk, UK, 1990-2008. Health Place.

[13] (2022). World Health Organization: Obesity and overweight. https://www.who.int/news-room/fact-sheets/detail/obesity-and-overweight.

[14] Kelly T, Yang W, Chen CS, Reynolds K, He J (2008). Global burden of obesity in 2005 and projections to 2030. Int J Obes (Lond).

[15] Saklayen MG (2018). The global epidemic of the metabolic syndrome. Curr Hypertens Rep.

[16] Laster J, Frame LA (2019). Beyond the calories-is the problem in the processing?. Curr Treat Options Gastroenterol.

[17] Ludwig DS, Ebbeling CB (2018). The carbohydrate-insulin model of obesity: beyond “calories in, calories out”. JAMA Intern Med.

[18] Howell S, Kones R (2017). "Calories in, calories out" and macronutrient intake: the hope, hype, and science of calories. Am J Physiol Endocrinol Metab.

[19] Schulte EM, Avena NM, Gearhardt AN (2015). Which foods may be addictive? The roles of processing, fat content, and glycemic load. PLoS One.

[20] Carter A, Hendrikse J, Lee N, Yücel M, Verdejo-Garcia A, Andrews ZB, Hall W (2016). The neurobiology of “food addiction” and its implications for obesity treatment and policy. Annu Rev Nutr.

[21] Polk SE, Schulte EM, Furman CR, Gearhardt AN (2017). Wanting and liking: separable components in problematic eating behavior?. Appetite.

[22] Rauber F, Chang K, Vamos EP, da Costa Louzada ML, Monteiro CA, Millett C, Levy RB (2021). Ultra-processed food consumption and risk of obesity: a prospective cohort study of UK Biobank. Eur J Nutr.

[23] Rauber F, Steele EM, Louzada ML, Millett C, Monteiro CA, Levy RB (2020). Ultra-processed food consumption and indicators of obesity in the United Kingdom population (2008-2016). PLoS One.

[24] Sung H, Park JM, Oh SU, Ha K, Joung H (2021). Consumption of ultra-processed foods increases the likelihood of having obesity in Korean women. Nutrients.

[25] Machado PP, Steele EM, Levy RB (2020). Ultra-processed food consumption and obesity in the Australian adult population. Nutr Diabetes.

[26] Juul F, Martinez-Steele E, Parekh N, Monteiro CA, Chang VW (2018). Ultra-processed food consumption and excess weight among US adults. Br J Nutr.

[27] Silva FM, Giatti L, de Figueiredo RC, Molina MD, de Oliveira Cardoso L, Duncan BB, Barreto SM (2018). Consumption of ultra-processed food and obesity: cross sectional results from the Brazilian Longitudinal Study of Adult Health (ELSA-Brasil) cohort (2008-2010). Public Health Nutr.

[28] Lustig RH (2020). Ultraprocessed food: addictive, toxic, and ready for regulation. Nutrients.

[29] Payab M, Kelishadi R, Qorbani M (2015). Association of junk food consumption with high blood pressure and obesity in Iranian children and adolescents: the CASPIAN-IV Study. J Pediatr (Rio J).

[30] Azemati B, Kelishadi R, Ahadi Z (2020). Association between junk food consumption and cardiometabolic risk factors in a national sample of Iranian children and adolescents population: the CASPIAN-V study. Eat Weight Disord.

[31] Albassam RS, Lei KY, Alnaami AM, Al-Daghri NM (2019). Correlations of neck circumference with body composition and cardiometabolic risk factors in Arab women. Eat Weight Disord.

[32] Gupta P, Shah D, Kumar P (2019). Indian Academy of Pediatrics guidelines on the fast and junk foods, sugar sweetened beverages, fruit juices, and energy drinks. Indian Pediatr.

[33] Chazelas E, Deschasaux M, Srour B (2020). Food additives: distribution and co-occurrence in 126,000 food products of the French market. Sci Rep.

[34] Banerjee A, Mukherjee S, Maji BK (2021). Worldwide flavor enhancer monosodium glutamate combined with high lipid diet provokes metabolic alterations and systemic anomalies: an overview. Toxicol Rep.

[35] Oplatowska-Stachowiak M, Elliott CT (2017). Food colors: existing and emerging food safety concerns. Crit Rev Food Sci Nutr.

[36] (2022). US Food and Drug Administration: Overview of food ingredients, additives & colors. https://www.fda.gov/food/food-ingredients-packaging/overview-food-ingredients-additives-colors.

[37] Suez J, Korem T, Zeevi D (2014). Artificial sweeteners induce glucose intolerance by altering the gut microbiota. Nature.

[38] Turner A, Veysey M, Keely S, Scarlett CJ, Lucock M, Beckett EL (2020). Intense sweeteners, taste receptors and the gut microbiome: a metabolic health perspective. Int J Environ Res Public Health.

[39] Rinninella E, Cintoni M, Raoul P, Gasbarrini A, Mele MC (2020). Food additives, gut microbiota, and irritable bowel syndrome: a hidden track. Int J Environ Res Public Health.

[40] Rinninella E, Cintoni M, Raoul P, Mora V, Gasbarrini A, Mele MC (2021). Impact of food additive titanium dioxide on gut microbiota composition, microbiota-associated functions, and gut barrier: a systematic review of in vivo animal studies. Int J Environ Res Public Health.

[41] Alting AC, van de Velde F (2012). Proteins as clean label ingredients in foods and beverages. Natural Food Additives, Ingredients and Flavorings.

[42] Center for Science in the Public Interest. Chemical Cuisaine. 1220 L St. NW Suite 300, Washington Washington, D.C D.C (2022). Center for Science in the Public Interest: Chemical cuisine ratings. https://www.cspinet.org/eating-healthy/chemical-cuisine.

[43] Ritz E, Hahn K, Ketteler M, Kuhlmann MK, Mann J (2012). Phosphate additives in food--a health risk. Dtsch Arztebl Int.

[44] Watanabe MT, Araujo RM, Vogt BP, Barretti P, Caramori JC (2016). Most consumed processed foods by patients on hemodialysis: alert for phosphate-containing additives and the phosphate-to-protein ratio. Clin Nutr ESPEN.

[45] Reddy AK, Ghoshal JA, Pk S, Trivedi GN, Ambareesha K (2021). Histomorphometric study on effects of monosodium glutamate in liver tissue of Wistar rats. J Basic Clin Physiol Pharmacol.

[46] Zanfirescu A, Ungurianu A, Tsatsakis AM (2019). A review of the alleged health hazards of monosodium glutamate. Compr Rev Food Sci Food Saf.

[47] Boldyrev AA, Bryushkova EA, Vladychenskaya EA (2012). NMDA receptors in immune competent cells. Biochemistry (Mosc).

[48] Hernández-Bautista RJ, Alarcón-Aguilar FJ, Del C Escobar-Villanueva M, Almanza-Pérez JC, Merino-Aguilar H, Fainstein MK, López-Diazguerrero NE (2014). Biochemical alterations during the obese-aging process in female and male monosodium glutamate (MSG)-treated mice. Int J Mol Sci.

[49] Hoang BX, Levine SA, Pham P, Shaw DG (2007). Hypothesis of the cause and development of neoplasms. Eur J Cancer Prev.

[50] Collison KS, Maqbool Z, Saleh SM (2009). Effect of dietary monosodium glutamate on trans fat-induced nonalcoholic fatty liver disease. J Lipid Res.

[51] Allison A, Fouladkhah A (2018). Adoptable interventions, human health, and food safety considerations for reducing sodium content of processed food products. Foods.

[52] Ni Mhurchu C, Capelin C, Dunford EK, Webster JL, Neal BC, Jebb SA (2011). Sodium content of processed foods in the United Kingdom: analysis of 44,000 foods purchased by 21,000 households. Am J Clin Nutr.

[53] Jaenke R, Barzi F, McMahon E, Webster J, Brimblecombe J (2017). Consumer acceptance of reformulated food products: a systematic review and meta-analysis of salt-reduced foods. Crit Rev Food Sci Nutr.

[54] Farquhar WB, Edwards DG, Jurkovitz CT, Weintraub WS (2015). Dietary sodium and health: more than just blood pressure. J Am Coll Cardiol.

[55] St-Jules DE, Jagannathan R, Gutekunst L, Kalantar-Zadeh K, Sevick MA (2017). Examining the proportion of dietary phosphorus from plants, animals, and food additives excreted in urine. J Ren Nutr.

[56] Haque M (2021). Fresh food is in struggle with processed: a global consternation. Adv Hum Biol.

[57] Dutta S, Haque M (2021). Taking highly palatable food or naively consuming fatal toxic diet. Bang J Med Sci.

[58] Sarnak MJ, Amann K, Bangalore S (2019). Chronic kidney disease and coronary artery disease: JACC state-of-the-art review. J Am Coll Cardiol.

[59] García Martín A, Varsavsky M, Cortés Berdonces M (2020). Phosphate disorders and clinical management of hypophosphatemia and hyperphosphatemia. Endocrinol Diabetes Nutr (Engl Ed).

[60] Masuda M, Yamamoto H, Takei Y (2020). All-trans retinoic acid reduces the transcriptional regulation of intestinal sodium-dependent phosphate co-transporter gene (Npt2b). Biochem J.

[61] Marcuccilli M, Chonchol M, Jovanovich A (2017). Phosphate binders and targets over decades: do we have it right now?. Semin Dial.

[62] Blaine J, Chonchol M, Levi M (2015). Renal control of calcium, phosphate, and magnesium homeostasis. Clin J Am Soc Nephrol.

[63] Stubbs J, Liu S, Quarles LD (2007). Role of fibroblast growth factor 23 in phosphate homeostasis and pathogenesis of disordered mineral metabolism in chronic kidney disease. Semin Dial.

[64] Institute of Medicine (US) Committee on Strategies to Reduce Sodium Intake ( 2010). Strategies to reduce sodium intake in the United States. The National Academies Collection: Reports funded by National Institutes of Health.

[65] Jüppner H (2011). Phosphate and FGF-23. Kidney Int Suppl.

[66] Ohnishi M, Razzaque MS (2010). Dietary and genetic evidence for phosphate toxicity accelerating mammalian aging. FASEB J.

[67] Kuro-o M (2011). Klotho and the aging process. Korean J Intern Med.

[68] Razzaque MS (2009). FGF23-mediated regulation of systemic phosphate homeostasis: is Klotho an essential player?. Am J Physiol Renal Physiol.

[69] Lorenzi O, Veyrat-Durebex C, Wollheim CB, Villemin P, Rohner-Jeanrenaud F, Zanchi A, Vischer UM (2010). Evidence against a direct role of klotho in insulin resistance. Pflugers Arch.

[70] Navarro-García JA, Fernández-Velasco M, Delgado C, Delgado JF, Kuro-O M, Ruilope LM, Ruiz-Hurtado G (2018). PTH, vitamin D, and the FGF-23-klotho axis and heart: going beyond the confines of nephrology. Eur J Clin Invest.

[71] Ha SK (2014). Dietary salt intake and hypertension. Electrolyte Blood Press.

[72] Coltherd JC, Staunton R, Colyer A (2019). Not all forms of dietary phosphorus are equal: an evaluation of postprandial phosphorus concentrations in the plasma of the cat. Br J Nutr.

[73] Sarki AM, Nduka CU, Stranges S, Kandala NB, Uthman OA (2015). Prevalence of hypertension in low- and middle-income countries: a systematic review and meta-analysis. Medicine (Baltimore).

[74] Grillo A, Salvi L, Coruzzi P, Salvi P, Parati G (2019). Sodium intake and hypertension. Nutrients.

[75] Geldsetzer P, Manne-Goehler J, Marcus ME (2019). The state of hypertension care in 44 low-income and middle-income countries: a cross-sectional study of nationally representative individual-level data from 1·1 million adults. Lancet.

[76] Moe SM, Zidehsarai MP, Chambers MA (2011). Vegetarian compared with meat dietary protein source and phosphorus homeostasis in chronic kidney disease. Clin J Am Soc Nephrol.

[77] Parpia AS, L'Abbé M, Goldstein M, Arcand J, Magnuson B, Darling PB (2018). The impact of additives on the phosphorus, potassium, and sodium content of commonly consumed meat, poultry, and fish products among patients with chronic kidney disease. J Ren Nutr.

[78] Shimada M, Shutto-Uchita Y, Yamabe H (2019). Lack of awareness of dietary sources of phosphorus is a clinical concern. In Vivo.

[79] Olanbiwonnu T, Holden RM (2018). Inorganic phosphate as a potential risk factor for chronic disease. CMAJ.

[80] Gonzalez-Parra E, Tuñón J, Egido J, Ortiz A (2012). Phosphate: a stealthier killer than previously thought?. Cardiovasc Pathol.

[81] Goh EV, Azam-Ali S, McCullough F, Roy Mitra S (2020). The nutrition transition in Malaysia; key drivers and recommendations for improved health outcomes. BMC Nutr.

[82] Baker P, Friel S (2014). Processed foods and the nutrition transition: evidence from Asia. Obes Rev.

[83] Baker P, Friel S (2016). Food systems transformations, ultra-processed food markets and the nutrition transition in Asia. Global Health.

[84] Paula Neto HA, Ausina P, Gomez LS, Leandro JG, Zancan P, Sola-Penna M (2017). Effects of food additives on immune cells as contributors to body weight gain and immune-mediated metabolic dysregulation. Front Immunol.

[85] Owen N, Healy GN, Matthews CE, Dunstan DW (2010). Too much sitting: the population health science of sedentary behavior. Exerc Sport Sci Rev.

[86] Ekelund U, Steene-Johannessen J, Brown WJ (2016). Does physical activity attenuate, or even eliminate, the detrimental association of sitting time with mortality? A harmonised meta-analysis of data from more than 1 million men and women. Lancet.

[87] Beaulieu K, Hopkins M, Blundell J, Finlayson G (2017). Impact of physical activity level and dietary fat content on passive overconsumption of energy in non-obese adults. Int J Behav Nutr Phys Act.

[88] Kartiko Sari I, Utari DM, Kamoshita S (2020). Increasing vegetable intake 400 g/day to control body weight and lipid profile in overweight hyperlipidemia menopausal women. J Public Health Res.

[89] Rosen ED, Spiegelman BM (2006). Adipocytes as regulators of energy balance and glucose homeostasis. Nature.

[90] Petersen MC, Shulman GI (2018). Mechanisms of insulin action and insulin resistance. Physiol Rev.

[91] Galicia-Garcia U, Benito-Vicente A, Jebari S (2020). Pathophysiology of type 2 diabetes mellitus. Int J Mol Sci.

[92] Rajesh Y, Sarkar D (2021). Association of adipose tissue and adipokines with development of obesity-induced liver cancer. Int J Mol Sci.

[93] Abdul-Ghani MA, Jayyousi A, DeFronzo RA, Asaad N, Al-Suwaidi J (2019). Insulin resistance the link between T2DM and CVD: basic mechanisms and clinical implications. Curr Vasc Pharmacol.

[94] Sinha S, Haque M (2022). Insulin resistance is cheerfully hitched with hypertension. Life (Basel).

[95] Cornier MA, Dabelea D, Hernandez TL (2008). The metabolic syndrome. Endocr Rev.

[96] Onat A (2011). Metabolic syndrome: nature, therapeutic solutions and options. Expert Opin Pharmacother.

[97] Prasad H, Ryan DA, Celzo MF, Stapleton D (2012). Metabolic syndrome: definition and therapeutic implications. Postgrad Med.

[98] Vasiljević J, Torkko JM, Knoch KP, Solimena M (2020). The making of insulin in health and disease. Diabetologia.

[99] Longo M, Zatterale F, Naderi J (2019). Adipose tissue dysfunction as determinant of obesity-associated metabolic complications. Int J Mol Sci.

[100] Hotamisligil GS, Shargill NS, Spiegelman BM (1993). Adipose expression of tumor necrosis factor-alpha: direct role in obesity-linked insulin resistance. Science.

[101] Alzamil H (2020). Elevated serum TNF-α is related to obesity in type 2 diabetes mellitus and is associated with glycemic control and insulin resistance. J Obes.

[102] Ullah MI, Alzahrani B, Alsrhani A, Atif M, Alameen AA, Ejaz H (2021). Determination of serum tumor necrosis factor-alpha (TNF-α) levels in metabolic syndrome patients from Saudi population. Pak J Med Sci.

[103] Ely BR, Clayton ZS, McCurdy CE, Pfeiffer J, Minson CT (2018). Meta-inflammation and cardiometabolic disease in obesity: can heat therapy help?. Temperature (Austin).

[104] Sinha S, Haque M (2022). Obesity is the alleyway to insulin resistance and type 2 diabetes mellitus. Adv Hum Biol.

[105] Esser N, Legrand-Poels S, Piette J, Scheen AJ, Paquot N (2014). Inflammation as a link between obesity, metabolic syndrome and type 2 diabetes. Diabetes Res Clin Pract.

[106] Kredel LI, Siegmund B (2014). Adipose-tissue and intestinal inflammation - visceral obesity and creeping fat. Front Immunol.

[107] Tsalamandris S, Antonopoulos AS, Oikonomou E (2019). The role of inflammation in diabetes: current concepts and future perspectives. Eur Cardiol.

[108] Bastard JP, Maachi M, Lagathu C (2006). Recent advances in the relationship between obesity, inflammation, and insulin resistance. Eur Cytokine Netw.

[109] Trayhurn P, Beattie JH (2001). Physiological role of adipose tissue: white adipose tissue as an endocrine and secretory organ. Proc Nutr Soc.

[110] Choe SS, Huh JY, Hwang IJ, Kim JI, Kim JB (2016). Adipose tissue remodeling: its role in energy metabolism and metabolic disorders. Front Endocrinol (Lausanne).

[111] Richard AJ, White U, Elks CM (2000). Adipose tissue: physiology to metabolic dysfunction. https://www.ncbi.nlm.nih.gov/books/NBK555602/.

[112] Chait A, den Hartigh LJ (2020). Adipose tissue distribution, inflammation and its metabolic consequences, including diabetes and cardiovascular disease. Front Cardiovasc Med.

[113] Panina YA, Yakimov AS, Komleva YK (2018). Plasticity of adipose tissue-derived stem cells and regulation of angiogenesis. Front Physiol.

[114] Li Y, Yun K, Mu R (2020). A review on the biology and properties of adipose tissue macrophages involved in adipose tissue physiological and pathophysiological processes. Lipids Health Dis.

[115] Boutens L, Hooiveld GJ, Dhingra S, Cramer RA, Netea MG, Stienstra R (2018). Unique metabolic activation of adipose tissue macrophages in obesity promotes inflammatory responses. Diabetologia.

[116] Castoldi A, Naffah de Souza C, Câmara NO, Moraes-Vieira PM (2015). The macrophage switch in obesity development. Front Immunol.

[117] Ellulu MS, Patimah I, Khaza'ai H, Rahmat A, Abed Y (2017). Obesity and inflammation: the linking mechanism and the complications. Arch Med Sci.

[118] Makki K, Froguel P, Wolowczuk I (2013). Adipose tissue in obesity-related inflammation and insulin resistance: cells, cytokines, and chemokines. ISRN Inflamm.

[119] de Luca C, Olefsky JM (2008). Inflammation and insulin resistance. FEBS Lett.

[120] Kim JH, Bachmann RA, Chen J (2009). Interleukin-6 and insulin resistance. Vitam Horm.

[121] McArdle MA, Finucane OM, Connaughton RM, McMorrow AM, Roche HM (2013). Mechanisms of obesity-induced inflammation and insulin resistance: insights into the emerging role of nutritional strategies. Front Endocrinol (Lausanne).

[122] Shi J, Fan J, Su Q, Yang Z (2019). Cytokines and abnormal glucose and lipid metabolism. Front Endocrinol (Lausanne).

[123] Kershaw EE, Flier JS (2004). Adipose tissue as an endocrine organ. J Clin Endocrinol Metab.

[124] de Oliveira Dos Santos AR, de Oliveira Zanuso B, Miola VF (2021). Adipokines, myokines, and hepatokines: crosstalk and metabolic repercussions. Int J Mol Sci.

[125] Galic S, Oakhill JS, Steinberg GR (2010). Adipose tissue as an endocrine organ. Mol Cell Endocrinol.

[126] Alipourfard I, Datukishvili N, Mikeladze D (2019). TNF-α downregulation modifies insulin receptor substrate 1 (IRS-1) in metabolic signaling of diabetic insulin-resistant hepatocytes. Mediators Inflamm.

[127] Blachnio-Zabielska AU, Hady HR, Markowski AR, Kurianiuk A, Karwowska A, Górski J, Zabielski P (2018). Inhibition of ceramide de novo synthesis affects adipocytokine secretion and improves systemic and adipose tissue insulin sensitivity. Int J Mol Sci.

[128] Saponaro C, Gaggini M, Carli F, Gastaldelli A (2015). The subtle balance between lipolysis and lipogenesis: a critical point in metabolic homeostasis. Nutrients.

[129] Yu YH, Ginsberg HN (2005). Adipocyte signaling and lipid homeostasis: sequelae of insulin-resistant adipose tissue. Circ Res.

[130] Zabielski P, Hady HR, Chacinska M, Roszczyc K, Gorski J, Blachnio-Zabielska AU (2018). The effect of high fat diet and metformin treatment on liver lipids accumulation and their impact on insulin action. Sci Rep.

[131] Cantley J, Ashcroft FM (2015). Q&A: insulin secretion and type 2 diabetes: why do β-cells fail?. BMC Biol.

[132] Han TS, Lean ME (2016). A clinical perspective of obesity, metabolic syndrome and cardiovascular disease. JRSM Cardiovasc Dis.

[133] Mendonça RD, Lopes AC, Pimenta AM, Gea A, Martinez-Gonzalez MA, Bes-Rastrollo M (2017). Ultra-processed food consumption and the incidence of hypertension in a Mediterranean cohort: the Seguimiento Universidad de Navarra project. Am J Hypertens.

[134] Shutto Y, Shimada M, Kitajima M, Yamabe H, Razzaque MS (2011). Lack of awareness among future medical professionals about the risk of consuming hidden phosphate-containing processed food and drinks. PLoS One.

[135] Chao A, Grilo CM, White MA, Sinha R (2014). Food cravings, food intake, and weight status in a community-based sample. Eat Behav.

[136] Alsiö J, Olszewski PK, Levine AS, Schiöth HB (2012). Feed-forward mechanisms: addiction-like behavioral and molecular adaptations in overeating. Front Neuroendocrinol.

[137] Berger J, Moller DE (2002). The mechanisms of action of PPARs. Annu Rev Med.

[138] Kurosu H, Yamamoto M, Clark JD (2005). Suppression of aging in mice by the hormone Klotho. Science.

[139] Goustin AS, Derar N, Abou-Samra AB (2013). Ahsg-fetuin blocks the metabolic arm of insulin action through its interaction with the 95-kD β-subunit of the insulin receptor. Cell Signal.

[140] Partridge D, Lloyd KA, Rhodes JM, Walker AW, Johnstone AM, Campbell BJ (2019). Food additives: assessing the impact of exposure to permitted emulsifiers on bowel and metabolic health - introducing the FADiets study. Nutr Bull.

[141] Chakraborty SP (2019). Patho-physiological and toxicological aspects of monosodium glutamate. Toxicol Mech Methods.

[142] Lerner A, Matthias T (2020). Processed food additive microbial transglutaminase and its cross-linked gliadin complexes are potential public health concerns in celiac disease. Int J Mol Sci.

[143] Hill MF, Bordoni B (2021). Hyperlipidemia. https://www.ncbi.nlm.nih.gov/books/NBK559182/.

[144] Shaman AM, Kowalski SR (2016). Hyperphosphatemia management in patients with chronic kidney disease. Saudi Pharm J.

[145] Nishime K, Sugiyama N, Okada K (2021). A new disease concept in the age of processed foods-phosphorus-burden disease; including CKD-MBD concrete analysis and the way to solution. Nutrients.

[146] Clark LT (2003). Treating dyslipidemia with statins: the risk-benefit profile. Am Heart J.

[147] Schaefer EJ, McNamara JR, Tayler T (2004). Comparisons of effects of statins (atorvastatin, fluvastatin, lovastatin, pravastatin, and simvastatin) on fasting and postprandial lipoproteins in patients with coronary heart disease versus control subjects. Am J Cardiol.

[148] Viegas CS, Santos L, Macedo AL (2018). Chronic kidney disease circulating calciprotein particles and extracellular vesicles promote vascular calcification: a role for GRP (GLA-rich protein). Arterioscler Thromb Vasc Biol.

[149] Stender S, Schuster H, Barter P, Watkins C, Kallend D (2005). Comparison of rosuvastatin with atorvastatin, simvastatin and pravastatin in achieving cholesterol goals and improving plasma lipids in hypercholesterolaemic patients with or without the metabolic syndrome in the MERCURY I trial. Diabetes Obes Metab.

[150] Carfagna F, Del Vecchio L, Pontoriero G, Locatelli F (2018). Current and potential treatment options for hyperphosphatemia. Expert Opin Drug Saf.

[151] Habbous S, Przech S, Acedillo R, Sarma S, Garg AX, Martin J (2017). The efficacy and safety of sevelamer and lanthanum versus calcium-containing and iron-based binders in treating hyperphosphatemia in patients with chronic kidney disease: a systematic review and meta-analysis. Nephrol Dial Transplant.

[152] Tonelli M, Sacks F, Pfeffer M, Gao Z, Curhan G (2005). Relation between serum phosphate level and cardiovascular event rate in people with coronary disease. Circulation.

[153] Laflamme D, Backus R, Brown S (2020). A review of phosphorus homeostasis and the impact of different types and amounts of dietary phosphate on metabolism and renal health in cats. J Vet Intern Med.

[154] Bhadada SK, Rao SD (2021). Role of phosphate in biomineralization. Calcif Tissue Int.

[155] Noori N, Sims JJ, Kopple JD, et a (2010). Organic and inorganic dietary phosphorus and its management in chronic kidney disease. Iran J Kidney Dis.

[156] Barreto FC, Barreto DV, Massy ZA, Drüeke TB (2019). Strategies for phosphate control in patients with CKD. Kidney Int Rep.

[157] McBride HM, Neuspiel M, Wasiak S (2006). Mitochondria: more than just a powerhouse. Curr Biol.

[158] Nguyen TT, Quan X, Xu S (2016). Intracellular alkalinization by phosphate uptake via type III sodium-phosphate cotransporter participates in high-phosphate-induced mitochondrial oxidative stress and defective insulin secretion. FASEB J.

[159] Nguyen TT, Quan X, Hwang KH (2015). Mitochondrial oxidative stress mediates high-phosphate-induced secretory defects and apoptosis in insulin-secreting cells. Am J Physiol Endocrinol Metab.

[160] Panov AV, Andreeva L, Greenamyre JT (2004). Quantitative evaluation of the effects of mitochondrial permeability transition pore modifiers on accumulation of calcium phosphate: comparison of rat liver and brain mitochondria. Arch Biochem Biophys.

[161] Bonora M, Pinton P (2014). The mitochondrial permeability transition pore and cancer: molecular mechanisms involved in cell death. Front Oncol.

[162] Brown RB, Razzaque MS (2015). Dysregulation of phosphate metabolism and conditions associated with phosphate toxicity. Bonekey Rep.

[163] Chen NX, Moe SM (2012). Vascular calcification: pathophysiology and risk factors. Curr Hypertens Rep.

[164] Lee SJ, Lee IK, Jeon JH (2020). Vascular calcification-new insights into its mechanism. Int J Mol Sci.

[165] Durham AL, Speer MY, Scatena M, Giachelli CM, Shanahan CM (2018). Role of smooth muscle cells in vascular calcification: implications in atherosclerosis and arterial stiffness. Cardiovasc Res.

[166] Zazzeroni L, Faggioli G, Pasquinelli G (2018). Mechanisms of arterial calcification: the role of matrix vesicles. Eur J Vasc Endovasc Surg.

[167] Cozzolino M, Ciceri P, Galassi A, Mangano M, Carugo S, Capelli I, Cianciolo G (2019). The key role of phosphate on vascular calcification. Toxins (Basel).

[168] Shobeiri N, Adams MA, Holden RM (2014). Phosphate: an old bone molecule but new cardiovascular risk factor. Br J Clin Pharmacol.

[169] Voelkl J, Lang F, Eckardt KU (2019). Signaling pathways involved in vascular smooth muscle cell calcification during hyperphosphatemia. Cell Mol Life Sci.

[170] Alesutan I, Luong TT, Schelski N (2021). Circulating uromodulin inhibits vascular calcification by interfering with pro-inflammatory cytokine signalling. Cardiovasc Res.

[171] Bao S, Guo Y, Diao Z, Guo W, Liu W (2020). Genome-wide identification of lncRNAs and mRNAs differentially expressed in human vascular smooth muscle cells stimulated by high phosphorus. Ren Fail.

[172] Chang Z, Yan G, Zheng J, Liu Z (2020). The lncRNA GAS5 inhibits the osteogenic differentiation and calcification of human vascular smooth muscle cells. Calcif Tissue Int.

[173] Bäck M, Aranyi T, Cancela ML (2018). Endogenous calcification inhibitors in the prevention of vascular calcification: a consensus statement from the cost action Eurosoftcalcnet. Front Cardiovasc Med.

[174] Alesutan I, Voelkl J, Feger M (2017). Involvement of vascular aldosterone synthase in phosphate-induced osteogenic transformation of vascular smooth muscle cells. Sci Rep.

[175] Hecht E, Freise C, Websky KV (2016). The matrix metalloproteinases 2 and 9 initiate uraemic vascular calcifications. Nephrol Dial Transplant.

[176] Luong TT, Schelski N, Boehme B (2018). Fibulin-3 attenuates phosphate-induced vascular smooth muscle cell calcification by inhibition of oxidative stress. Cell Physiol Biochem.

[177] Freise C, Kretzschmar N, Querfeld U (2016). Wnt signaling contributes to vascular calcification by induction of matrix metalloproteinases. BMC Cardiovasc Disord.

[178] Sena BF, Figueiredo JL, Aikawa E (2017). Cathepsin S as an inhibitor of cardiovascular inflammation and calcification in chronic kidney disease. Front Cardiovasc Med.

[179] Jiang W, Zhang Z, Li Y (2021). The cell origin and role of osteoclastogenesis and osteoblastogenesis in vascular calcification. Front Cardiovasc Med.

[180] Takashi Y, Fukumoto S (2020). Phosphate-sensing and regulatory mechanism of FGF23 production. J Endocrinol Invest.

[181] Singh AP, Sosa MX, Fang J (2019). Αklotho regulates age-associated vascular calcification and lifespan in zebrafish. Cell Rep.

[182] Ohnishi M, Kato S, Razzaque MS (2011). Genetic induction of phosphate toxicity significantly reduces the survival of hypercholesterolemic obese mice. Biochem Biophys Res Commun.

[183] Lampila LE (2013). Applications and functions of food-grade phosphates. Ann N Y Acad Sci.

[184] Erem S, Razzaque MS (2018). Dietary phosphate toxicity: an emerging global health concern. Histochem Cell Biol.

[185] Ali FN, Falkner B, Gidding SS, Price HE, Keith SW, Langman CB (2014). Fibroblast growth factor-23 in obese, normotensive adolescents is associated with adverse cardiac structure. J Pediatr.

[186] Kutluturk Y, Akinci A, Ozerol IH, Yologlu S (2019). The relationship between serum FGF-23 concentration and insulin resistance, prediabetes and dyslipidemia in obese children and adolescents. J Pediatr Endocrinol Metab.

[187] Brown RB, Haq A, Stanford CF, Razzaque MS (2015). Vitamin D, phosphate, and vasculotoxicity. Can J Physiol Pharmacol.

[188] Wang PW, Pang Q, Zhou T, Song XY, Pan YJ, Jia LP, Zhang AH (2022). Irisin alleviates vascular calcification by inhibiting VSMC osteoblastic transformation and mitochondria dysfunction via AMPK/Drp1 signaling pathway in chronic kidney disease. Atherosclerosis.

[189] Zhang D, Bi X, Liu Y (2017). High phosphate-induced calcification of vascular smooth muscle cells is associated with the TLR4/NF-κb signaling pathway. Kidney Blood Press Res.

[190] Sealand R, Razavi C, Adler RA (2013). Diabetes mellitus and osteoporosis. Curr Diab Rep.

[191] Sergienko EA, Millán JL (2010). High-throughput screening of tissue-nonspecific alkaline phosphatase for identification of effectors with diverse modes of action. Nat Protoc.

[192] Hénaut L, Candellier A, Boudot C (2019). New insights into the roles of monocytes/macrophages in cardiovascular calcification associated with chronic kidney disease. Toxins (Basel).

[193] Disthabanchong S (2018). Phosphate and cardiovascular disease beyond chronic kidney disease and vascular calcification. Int J Nephrol.

[194] Lino M, Wan MH, Rocca AS (2018). Diabetic vascular calcification mediated by the collagen receptor Discoidin Domain Receptor 1 via the phosphoinositide 3-kinase/Akt/runt-related transcription factor 2 signaling axis. Arterioscler Thromb Vasc Biol.

[195] Jin H, Xu CX, Lim HT (2009). High dietary inorganic phosphate increases lung tumorigenesis and alters Akt signaling. Am J Respir Crit Care Med.

[196] Jüppner H, Wolf M, Salusky IB (2010). FGF-23: more than a regulator of renal phosphate handling?. J Bone Miner Res.

[197] Kuro-o M (2013). Klotho, phosphate and FGF-23 in ageing and disturbed mineral metabolism. Nat Rev Nephrol.

[198] Hamed SA (2019). Neurologic conditions and disorders of uremic syndrome of chronic kidney disease: presentations, causes, and treatment strategies. Expert Rev Clin Pharmacol.

[199] Lips P, Eekhoff M, van Schoor N, Oosterwerff M, de Jongh R, Krul-Poel Y, Simsek S (2017). Vitamin D and type 2 diabetes. J Steroid Biochem Mol Biol.

[200] Alamdari A, Mozafari R, Tafakhori A (2015). An inverse association between serum vitamin D levels with the presence and severity of impaired nerve conduction velocity and large fiber peripheral neuropathy in diabetic subjects. Neurol Sci.

[201] Motori E, Atanassov I, Kochan SM (2020). Neuronal metabolic rewiring promotes resilience to neurodegeneration caused by mitochondrial dysfunction. Sci Adv.

[202] Miyamoto K, Haito-Sugino S, Kuwahara S (2011). Sodium-dependent phosphate cotransporters: lessons from gene knockout and mutation studies. J Pharm Sci.

[203] Razzaque MS (2014). Bone-kidney axis in systemic phosphate turnover. Arch Biochem Biophys.

[204] Gattineni J, Bates C, Twombley K (2009). FGF23 decreases renal NaPi-2a and NaPi-2c expression and induces hypophosphatemia in vivo predominantly via FGF receptor 1. Am J Physiol Renal Physiol.

[205] Liu S, Tang W, Zhou J, Stubbs JR, Luo Q, Pi M, Quarles LD (2006). Fibroblast growth factor 23 is a counter-regulatory phosphaturic hormone for vitamin D. J Am Soc Nephrol.

[206] Xu M, Li H, Bai Y (2021). miR-129 blocks secondary hyperparathyroidism-inducing FGF23/αklotho signaling in mice with chronic kidney disease. Am J Med Sci.

[207] Lanske B, Razzaque MS (2007). Premature aging in klotho mutant mice: cause or consequence?. Ageing Res Rev.

[208] Palmer SC, Hayen A, Macaskill P, Pellegrini F, Craig JC, Elder GJ, Strippoli GF (2011). Serum levels of phosphorus, parathyroid hormone, and calcium and risks of death and cardiovascular disease in individuals with chronic kidney disease: a systematic review and meta-analysis. JAMA.

[209] Kuro-O M (2013). A phosphate-centric paradigm for pathophysiology and therapy of chronic kidney disease. Kidney Int Suppl (2011).

[210] Jha V, Garcia-Garcia G, Iseki K (2013). Chronic kidney disease: global dimension and perspectives. Lancet.

[211] Uribarri J (2013). Dietary phosphorus and kidney disease. Ann N Y Acad Sci.

[212] He P, Mann-Collura O, Fling J, Edara N, Hetz R, Razzaque MS (2021). High phosphate actively induces cytotoxicity by rewiring pro-survival and pro-apoptotic signaling networks in HEK293 and HeLa cells. FASEB J.

[213] Shutto Y, Shimada M, Kitajima M, Yamabe H, Saitoh Y, Saitoh H, Razzaque MS (2013). Inadequate awareness among chronic kidney disease patients regarding food and drinks containing artificially added phosphate. PLoS One.

[214] Anyanwu OA, Folta SC, Zhang FF, Chui K, Chomitz VR, Kartasurya MI, Naumova EN (2022). A cross-sectional assessment of dietary patterns and their relationship to hypertension and obesity in Indonesia. Curr Dev Nutr.

[215] Almandoz JP, Xie L, Schellinger JN (2022). Changes in body weight, health behaviors, and mental health in adults with obesity during the COVID-19 pandemic. Obesity (Silver Spring).

[216] Rey-García J, Donat-Vargas C, Sandoval-Insausti H (2021). Ultra-processed food consumption is associated with renal function decline in older adults: a prospective cohort study. Nutrients.

[217] Sandoval-Insausti H, Jiménez-Onsurbe M, Donat-Vargas C, Rey-García J, Banegas JR, Rodríguez-Artalejo F, Guallar-Castillón P (2020). Ultra-processed food consumption is associated with abdominal obesity: a prospective cohort study in older adults. Nutrients.

[218] Beslay M, Srour B, Méjean C (2020). Ultra-processed food intake in association with BMI change and risk of overweight and obesity: a prospective analysis of the French NutriNet-Santé cohort. PLoS Med.

[219] Donat-Vargas C, Sandoval-Insausti H, Rey-García J (2021). High consumption of ultra-processed food is associated with incident dyslipidemia: a prospective study of older adults. J Nutr.

[220] Llavero-Valero M, Escalada-San Martín J, Martínez-González MA, Basterra-Gortari FJ, de la Fuente-Arrillaga C, Bes-Rastrollo M (2021). Ultra-processed foods and type-2 diabetes risk in the SUN project: a prospective cohort study. Clin Nutr.

[221] Montero-Salazar H, Donat-Vargas C, Moreno-Franco B, Sandoval-Insausti H, Civeira F, Laclaustra M, Guallar-Castillón P (2020). High consumption of ultra-processed food may double the risk of subclinical coronary atherosclerosis: the Aragon Workers' Health Study (AWHS). BMC Med.

[222] Du S, Kim H, Rebholz CM (2021). Higher ultra-processed food consumption is associated with increased risk of incident coronary artery disease in the Atherosclerosis Risk in Communities study. J Nutr.

[223] Kurniawan AL, Hsu CY, Rau HH, Lin LY, Chao JC (2019). Association of kidney function-related dietary pattern, weight status, and cardiovascular risk factors with severity of impaired kidney function in middle-aged and older adults with chronic kidney disease: a cross-sectional population study. Nutr J.

[224] Kurniawan AL, Hsu CY, Lee HA, Rau HH, Paramastri R, Syauqy A, Chao JC (2020). Comparing two methods for deriving dietary patterns associated with risk of metabolic syndrome among middle-aged and elderly Taiwanese adults with impaired kidney function. BMC Med Res Methodol.

[225] Vogelzangs N, van der Kallen CJ, van Greevenbroek MM (2020). Metabolic profiling of tissue-specific insulin resistance in human obesity: results from the Diogenes study and the Maastricht Study. Int J Obes (Lond).

[226] Wang T, Lu J, Shi L (2020). Association of insulin resistance and β-cell dysfunction with incident diabetes among adults in China: a nationwide, population-based, prospective cohort study. Lancet Diabetes Endocrinol.

[227] Niu Y, Tang Q, Zhao X (2021). Obesity-induced insulin resistance is mediated by high uric acid in obese children and adolescents. Front Endocrinol (Lausanne).

[228] Martins AM, Bello Moreira AS, Canella DS (2017). Elderly patients on hemodialysis have worse dietary quality and higher consumption of ultraprocessed food than elderly without chronic kidney disease. Nutrition.

[229] Gutiérrez OM (2013). Sodium- and phosphorus-based food additives: persistent but surmountable hurdles in the management of nutrition in chronic kidney disease. Adv Chronic Kidney Dis.

[230] Nadkarni GN, Uribarri J (2014). Phosphorus and the kidney: what is known and what is needed. Adv Nutr.

[231] Calvo MS, Uribarri J (2013). Public health impact of dietary phosphorus excess on bone and cardiovascular health in the general population. Am J Clin Nutr.

[232] Calvo MS, Moshfegh AJ, Tucker KL (2014). Assessing the health impact of phosphorus in the food supply: issues and considerations. Adv Nutr.

[233] Manna P, Jain SK (2015). Obesity, oxidative stress, adipose tissue dysfunction, and the associated health risks: causes and therapeutic strategies. Metab Syndr Relat Disord.

[234] Fernández-Sánchez A, Madrigal-Santillán E, Bautista M (2011). Inflammation, oxidative stress, and obesity. Int J Mol Sci.

[235] Fonseca-Alaniz MH, Takada J, Alonso-Vale MI, Lima FB (2007). Adipose tissue as an endocrine organ: from theory to practice. J Pediatr (Rio J).

[236] Montezano AC, Touyz RM (2012). Reactive oxygen species and endothelial function--role of nitric oxide synthase uncoupling and Nox family nicotinamide adenine dinucleotide phosphate oxidases. Basic Clin Pharmacol Toxicol.

[237] Evans-Molina C, Hatanaka M, Mirmira RG (2013). Lost in translation: endoplasmic reticulum stress and the decline of β-cell health in diabetes mellitus. Diabetes Obes Metab.

[238] Zhao MM, Xu MJ, Cai Y (2011). Mitochondrial reactive oxygen species promote p65 nuclear translocation mediating high-phosphate-induced vascular calcification in vitro and in vivo. Kidney Int.

[239] Ertuglu LA, Afsar B, Yildiz AB, Demiray A, Ortiz A, Covic A, Kanbay M (2021). Substitution of sugar-sweetened beverages for other beverages: can it be the next step towards healthy aging?. Curr Nutr Rep.

[240] Razzaque MS (2011). Phosphate toxicity: new insights into an old problem. Clin Sci (Lond).

[241] Nakatani T, Ohnishi M, Razzaque MS (2009). Inactivation of klotho function induces hyperphosphatemia even in presence of high serum fibroblast growth factor 23 levels in a genetically engineered hypophosphatemic (Hyp) mouse model. FASEB J.

[242] Fukagawa M, Hamada Y, Nakanishi S, Tanaka M (2006). The kidney and bone metabolism: nephrologists' point of view. J Bone Miner Metab.

[243] Olauson H, Vervloet MG, Cozzolino M, Massy ZA, Ureña Torres P, Larsson TE (2014). New insights into the FGF23-Klotho axis. Semin Nephrol.

[244] Tsuchiya K, Nagano N, Nitta K (2015). Klotho/FGF23 Axis in CKD. Contrib Nephrol.

